# Economic Impact of Gene Expression Profiling in Patients with Early-Stage Breast Cancer in France

**DOI:** 10.1371/journal.pone.0128880

**Published:** 2015-06-18

**Authors:** Gregory Katz, Olivier Romano, Cyril Foa, Anne-Lise Vataire, Jean-Victor Chantelard, Robert Hervé, Hugues Barletta, Axel Durieux, Jean-Pierre Martin, Rémy Salmon

**Affiliations:** 1 ESSEC Business School, Chair of Therapeutic Innovation, Paris, France and Singapore; 2 Générale de Santé, Hôpital Privé la Louvière, Lille, France; 3 Générale de Santé, Hôpital Privé Clairval, Marseille, France; 4 Creativ-Ceutical, Paris, France; 5 Générale de Santé, Hôpital Privé Paul d’Egine, Champigny-sur-Marne, France; 6 Générale de Santé, Hôpital Privé Drôme-Ardèche, Guilherand-Granges, France; 7 Générale de Santé, Hôpital Privé des Peupliers, Paris, France; 8 Générale de Santé, Hôpital Privé Jean Mermoz, Lyon, France; 9 Fondation Générale de Santé, Paris, France; 10 Générale de Santé, Hôpital Privé Villeneuve-d’Ascq, Villeneuve-d’Ascq, France; 11 Université Lyon Claude Bernard, Lyon, France; Queen Mary Hospital, HONG KONG

## Abstract

**Background and Aims:**

The heterogeneous nature of breast cancer can make decisions on adjuvant chemotherapy following surgical resection challenging. Onco*type* DX is a validated gene expression profiling test that predicts the likelihood of adjuvant chemotherapy benefit in early-stage breast cancer. The aim of this study is to determine the costs of chemotherapy in private hospitals in France, and evaluate the cost-effectiveness of Onco*type* DX from national insurance and societal perspectives.

**Methods:**

A multicenter study was conducted in seven French private hospitals, capturing retrospective data from 106 patient files. Cost estimates were used in conjunction with a published Markov model to assess the cost-effectiveness of using Onco*type* DX to inform chemotherapy decision making versus standard care. Sensitivity analyses were performed.

**Results:**

The cost of adjuvant chemotherapy in private hospitals was estimated at EUR 8,218 per patient from a national insurance perspective and EUR 10,305 from a societal perspective. Cost-effectiveness analysis indicated that introducing Onco*type* DX improved life expectancy (+0.18 years) and quality-adjusted life expectancy (+0.17 QALYs) versus standard care. Onco*type* DX was found cost-effective from a national insurance perspective (EUR 2,134 per QALY gained) and cost saving from a societal perspective versus standard care. Inclusion of lost productivity costs in the modeling analysis meant that costs for eligible patients undergoing Onco*type* DX testing were on average EUR 602 lower than costs for those receiving standard care.

**Conclusions:**

As Onco*type* DX was found both cost and life-saving from a societal perspective, the test was considered to be dominant to standard care. However, the delay in coverage has the potential to erode the quality of the French healthcare system, thus depriving patients of technologies that could improve clinical outcomes and allow healthcare professionals to better allocate hospital resources to improve the standard of care for all patients.

## Introduction

Breast cancer is the second most common cancer in the world and, by far, the most frequently occurring cancer in women with an estimated 1.67 million new cases diagnosed in 2012 (resulting in 522,000 deaths).[1] The figures represent approximately one in four of all cancer cases.[1] Approximately 54,000 new cases were diagnosed in France in 2012, representing 33% of all new cancer diagnoses in women.[2, 3] Encouragingly, however, screening practices now mean that most cases are identified at an early stage and, as a result, the utilization of adjuvant chemotherapy following surgical resection has increased in recent years.

Breast cancer is a heterogeneous disease and prognosis, survival and recurrence rates can vary widely. They are influenced by a number of factors including disease stage at diagnosis (based on tumor size, lymph node involvement and distant metastases), presence of particular molecular markers including, in particular, the estrogen and progesterone receptors (ER and PR, respectively) and the human epidermal growth factor receptor 2 (HER2). Decisions on whether to use adjuvant chemotherapy in patients with early, invasive, operable breast cancer have traditionally relied on such clinical, pathologic and biological markers. However, these indicators are imperfect in terms of reproducibility and lack of standardization (for example with the Ki-67 marker), leaving room for interpretation. Current estimates indicate that more than 60% of patients with hormone receptor positive breast cancer receive adjuvant chemotherapy. However several studies have demonstrated that only 4–5% of patients are likely to benefit from chemotherapy.[4] Further, chemotherapy is often associated with many short and long-term side effects, leading to significant costs and serious psychological sequelae for patients, such as anxiety and emotional distress.[5] In addition, chemotherapy can have an impact on the patient’s professional life. Evidence indicates that absenteeism from work is twice as high in women receiving chemotherapy than in those who do not, and can lead to early retirement in some cases.[6] Lost productivity has been estimated to make up more than one-quarter of the total costs of chemotherapy for breast cancer, making it the single biggest cost component, and an important part of any cost evaluation.[7]

Gene expression profiling and immunohistochemistry tests aim to improve the targeting of chemotherapy in breast cancer by more accurately identifying those patients who will benefit most, based on knowledge of biologic features of cancer that indicate increased likelihood of rapid growth, recurrences and/or metastasis. The Onco*type* DX Breast Cancer Test (Genomic Health Inc., Redwood City, CA, USA), is one such assay and has been shown to successfully predict the likelihood of chemotherapy benefit as well as distant recurrence 10 years after diagnosis in patients with early-stage, node-negative and node-positive ER-positive breast cancer. The validity of Onco*type* DX has been demonstrated in a number of clinical studies both for prognosis and prediction of likelihood of chemotherapy benefit.[8–12] A number of studies evaluating the impact of the assay on adjuvant therapy decisions in patients with ER+ early breast cancer have demonstrated that knowledge of the Onco*type* DX Recurrence Score (RS) affects management of patients. Importantly, the studies reveal that every second patient originally recommended adjuvant chemotherapy plus endocrine treatment is recommended endocrine treatment alone after knowledge of the RS.[13–16] Although not currently reimbursed in France, the National Institute for Health and Care Excellence (NICE) in the UK indicated that Onco*type* DX has the most robust evidence of the tests currently available for breast cancer.[17] Its use to inform decision making in adjuvant chemotherapy is supported by several guidelines on best clinical practice, including those from European Society for Medical Oncology (ESMO), the American Society of Clinical Oncology (ASCO), the National Comprehensive Cancer Network (NCCN), and St. Gallen.[18–21]

In 2012, a cost-effectiveness evaluation was published that investigated the health economic implications of introducing the Onco*type* DX in public hospitals in France.[22] However, there is a paucity of data on the costs of chemotherapy and the cost-effectiveness of introducing gene expression profiling or expanded immunohistochemistry tests in private hospitals in the French setting. This is of particular concern given that, in 2012, private hospitals performed 43% of surgical interventions for breast cancer and 28% of chemotherapies for all cancers.[23] The aims of the present study, therefore, were two-fold: to evaluate the cost of adjuvant chemotherapy for early stage breast cancer from societal and national insurance perspectives in private hospitals, and to perform a cost-effectiveness evaluation investigating the use of Onco*type* DX to guide chemotherapy decision making versus standard approaches in eligible patients with early-stage breast cancer in private hospitals in France.

## Materials and Methods

### Cost of chemotherapy

A retrospective database analysis was performed to evaluate both direct and indirect costs associated with adjuvant chemotherapy in women with ER+, HER2−, early-stage breast cancer. This study was submitted and approved by the French ethics committee and a National Institutional Review Board (CPP Ile de France 3). Retrospective data from 106 patient files were anonymized and de-identified prior to analysis. Patient records were collected from seven private hospitals belonging to the group Générale de Santé in France: Hôpital Privé La Louvière (Lille), Hôpital Privé Villeneuve-d’Ascq (Villeneuve-d’Ascq), Hôpital Privé Hôpital Jean Mermoz (Lyon), Hôpital Privé Clairval (Marseille), Hôpital Privé des Peupliers (Paris), Hôpital Privé Drôme Ardèche (Guilherand-Granges) and Hôpital Privé Paul d’Egine (Champigny-sur-Marne). Resource data were extracted from medical records of female patients who have undergone surgery for breast cancer from January 2008 to January 2013. Inclusion criteria were: 1) women who received all cycles of chemotherapy within the same private hospital, and 2) women with ER+, negative HER2 status and no node involvement. Patients with incomplete medical files were excluded. Data were collected from the start of therapy (including the pre-chemotherapy period) to the end of adjuvant chemotherapy treatment (Table 1). Costs associated with other interventions such as radiotherapy and other treatments such as endocrine therapy were not included.

**Table 1:** Data collected for the retrospective analysis of chemotherapy costs.

Time period	Information collected
Baseline information of patient characteristics and pre-chemotherapy procedures	**Patient characteristics**
	Age
	Socio-professional group
	Body weight
	Height
	Body surface area
	TNM (tumor, node, metastasis) classification
	Ki-67 status
	HER2 status
	ER and PR status
	**Pre-chemotherapy tests and procedures**
	Central venous access implantation
	Electrocardiogram
	Laboratory tests
	Functional heart tests
	Oncologist consultations
Information collected for each chemotherapy cycle	Chemotherapy regimen (chemotherapy agents, cumulative dose, start date, and end date)
	Prophylactic agents (prophylactic treatment and cumulative dose)
	Side effects (medications taken including hospitalizations and consultations)
	Visits (number of general practitioner and specialist visits)
	Hospitalizations (start date, end date, admission service, reason of admission, type of admission (day care or complete hospitalization) link to adverse events
	Laboratory tests (hospital, home or laboratory, list of items tested)
	Home care (date and reason)
	Transport (ambulance, taxi, personal car, patient transport service ambulance, public transport, voucher from social insurance, number of kilometers between home and hospital)
	Sick leave (start date and end date)

Costs were estimated in 2013 Euros (EUR) using the French tariff system T2A (“tarification à l'activité”). Fixed T2A payments were recorded covering drug costs (EUR 25), excluding expensive products, and administration costs (EUR 272.48), using the results of the national cost scale (Echelle Nationale de Coûts, 2011) defining T2A payments.[23] Costs associated with expensive medications not covered by the T2A tariff system were added separately. Physician consultations and medication costs were extracted from lists of tariffs available from the national health insurance system (Caisse Nationale de l'Assurance Maladie). Hospitalisation costs were determined using the “Groupe Homogène de Malades” (GHM codes are analogous to DRG codes) and the corresponding unit costs from the national scale of costs.[23] Medication costs were based on daily or cumulative dosage and the package size with the least expensive unit cost was used wherever possible. Treatment related to prophylactic or symptomatic prescriptions could be delivered by hospitals or pharmacy. Missing values were imputed for cumulative/daily doses using another prescription of the study for the same treatment or from standard prescription, recommended by ANSM (Agence National de Sécurité du Médicament et des produits de santé).[24] The cost of administration of growth factors by nurses at the patient’s home included an injection fee and compensation for the nurse travel cost, and assumed an average distance of 6.8 km.[25] Cancer is categorized as a long-term condition (“affection longue durée”) for which patients are entitled to 100% reimbursement of healthcare costs. It was assumed that patients received transport vouchers, reimbursed by national insurance, for taxi, ambulance or patient transport service.

A human capital approach was used to estimate costs associated with lost workplace productivity based on sick leave payments from the national health insurance system. Costs were estimated as the number of sick-leave days multiplied by the average daily salary for women (EUR 60.53 per day in 2012).[26] In cases of missing values, lost productivity costs were imputed based on existing data. For presentation, costs were categorized as follows: monitoring costs (consultation, laboratory tests and pre-chemotherapy), drug and administration costs (prophylactic prescriptions and chemotherapy management), side effect costs (drug-related hospitalizations and consultations), and transport and absenteeism costs.

### Cost-effectiveness analysis

Cost-effectiveness analysis was performed using a published Markov model designed to compare the cost-effectiveness of using the Onco*type* DX Recurrence Score (RS) to guide chemotherapy decision making with standard care.[22] The proportions of patients recommended adjuvant chemotherapy using either standard care or the Onco*type* DX RS were derived from a meta-analysis of nine international studies on the decision impact associated with Onco*type* DX testing.[13] The meta-analysis showed reductions in utilization of chemotherapy of 22% and 7.5% for patients with low risk and intermediate risk of recurrence, respectively. For patients with a high risk of recurrence, Onco*type* DX testing led to an increase of 5.6% in the prescribing of chemotherapy relative to standard care. Based on this therapy allocation, the model made projections of long-term costs and clinical outcomes based on landmark data from a prospective clinical trial comparing chemotherapy and hormonal therapy or hormone therapy alone (National Surgical Adjuvant Breast and Bowel Project [NSABP] B20 trial).[8] Three-year survival after metastatic recurrence of breast cancer came from a prospective study conducted in three French hospitals between 2001 and 2006 (mean 35.8 months, 95% confidence interval 31.7 to 39.1 months).[27] Overall mortality rates were obtained from the French National Demographic Institute.[28]

The analysis was performed from the “collective perspective” defined by the Haute Autorité de Santé (HAS), which includes all direct costs by the national insurance, private insurance or patients. Costs for the Onco*type* DX test were based on the list price (EUR 3,180). Estimates of costs associated with chemotherapy were obtained from the retrospective study described above. The cost of distant recurrence events was based on the data published by Bonneterre *et al*. in 2005.[29] Other model inputs were consistent with the previously published cost-effectiveness analysis of Onco*type* DX in the French setting.[22] Both life expectancy and quality-adjusted life expectancy were evaluated by the model. Published utility scores (consistent with previous cost-effectiveness analyses of Onco*type* DX were used to estimate quality-adjusted life expectancy, with chemotherapy associated with a utility decrement of 0.07 and annual utility scores of 0.60 and 0.78 for patients with and without recurrence, respectively.[30–32]

The base case time horizon was set to 30 years (the model had a one year cycle length) to capture long-term recurrence risk. Future costs and clinical benefits were discounted at 4% *per annum* in line with published recommendations.[33] Probabilistic sensitivity analysis (PSA) was performed by performing 1,000 iterations of the base case analysis with sampling from distributions around patient age, allocation of adjuvant therapy, recurrence rates, post recurrence survival, costs of the test, chemotherapy and recurrence, and health-related quality of life utilities. Multiple one-way sensitivity analyses were performed to identify key drivers of model outcomes. Each input parameter was varied between the upper and lower confidence interval as published. For those parameters where no confidence interval was available, the input value was varied by +/− 25%.

## Results

### Cost of adjuvant chemotherapy in early-stage breast cancer

A total of 106 patients were included in the retrospective analysis of chemotherapy costs, with a mean age of 53.2 years (Table 2 and S1 File). The most frequently prescribed treatment protocol (47.5% of all cycles) in the private hospital setting was a combination of three cycles of epirubicine, cyclophosphamide and fluorouracil (5-FU) followed by three cycles of docetaxel (27.6% of cycles). For managing side effects, the most commonly prescribed agents were anti-emetics (92% of cycles) and growth factors (58% of cycles). Over the 577 cycles reported, a total of 32 re-hospitalizations occurred involving 19% of patients, primarily in surgical wards (25%) and medicine services (25%).

**Table 2:** Summary of patient characteristics in the chemotherapy costing analysis.

	N	Mean (standard deviation)
**Age (years)**	106	53.2 (11.3)
**Height (cm)**	106	161.4 (7.4)
**Weight (kg)**	106	64.9 (13.4)
**Surface area (m^2^)**	106	1.7 (0.2)
**Working status (%)**	92	
Working		62.1
Not working		10.8
Retired		27.1
**Tumor stage (%)**	100	
1		50.0
2		50.0
**TNM staging (%)**	106	
T1n0		49.0
T2n0		47.1
T3n0		3.7
HER2 negative (%)	106	100
ER positive (%)	106	0
PR positive (%)	106	83.1
**Chemotherapy cycles under protocol (%)**	106	
Docetaxel		27.6
Docetaxel + C		13.5
Docetaxel + C + E		3.1
Doxorubicine + C		2.6
Epirubicine		0.2
Epirubicine + C		3.3
Epirubicine + C + 5FU		47.5
Epirubicine + 5FU		0.2
Paclitaxel		1.7
Paclitaxel + C		0.3

C, cyclophosphamide; E, epirubicin; 5FU, fluorouracil; HER, human epidermal growth factor receptor; ER, estrogen receptor; PR, progesterone receptor.

The analysis demonstrated that the mean cost of chemotherapy in the private hospital setting was approximately EUR 8,218 from a healthcare payer perspective (direct costs only) (Table 3). Approximately 62% of the study population were employed (Table 2) and 90.7% of these patients took sick leave during the chemotherapy treatment period. Factoring this lost productivity into the analysis showed that the mean costs of adjuvant chemotherapy were EUR 10,305 from a societal perspective (Table 3). Absenteeism was the main driver of costs from both the healthcare payer and societal perspectives (Table 3), accounting for approximately 24% and 39% of total costs, respectively. The other main cost driver was prophylactic prescriptions, which contributed 30% of payer costs and 24% of total costs from the societal perspective.

**Table 3:** Cost of adjuvant chemotherapy by components for societal and payer perspective.

		Mean cost (standard deviation) [EUR]	
	N	Payer perspective	Societal perspective
Administration chemotherapy	106	1,549.81 (385.79)	1,549.81 (385.79)
Chemotherapy drug	106	302.69 (85.35)	302.69 (85.35)
Prophylactic prescription	106	2,440.39 (2249.97)	2,440.39 (2249.97)
Side effect management	106	687.64 (2192.31)	687.64 (2192.31)
Monitoring	106	616.62 (147.33)	616.62 (147.33)
Transport	106	624.84 (512.32)	714.46 (608.48)
Absenteeism	106	1,996.37 (1724.38)	3,993.39 (3449.32)
Total (including absenteeism)	106	8,218.37 (3784.40)	10,305.01 (4,979.21)
Total (without absenteeism)	106	6,222.00 (3181.09)	6,311.62 (3,215.39)

All costs are expressed in 2013 Euros (EUR).

### Cost-effectiveness analysis of Onco*type* DX to guide chemotherapy decision making

Using Onco*type* DX to guide adjuvant chemotherapy decision making was associated with improvements in both life expectancy and quality-adjusted life expectancy compared with standard care (Table 4). Using the Onco*type* DX RS was associated with an improvement in mean discounted life expectancy of approximately 0.18 years versus standard care in eligible patients, due primarily to more appropriate allocation of chemotherapy for high risk patients. When the analysis captured health-related quality of life, Onco*type* DX was associated with a benefit of approximately 0.17 QALY versus standard care, due to chemotherapy sparing in low risk patients in addition to survival benefits in high risk patients.

**Table 4:** Summary cost-effectiveness results for Onco*type* DX versus standard care to inform adjuvant chemotherapy decision making in French private hospitals.

	Onco*type* DX	Standard care	Difference
Life expectancy (years)	14.60	14.42	+0.18
Quality-adjusted life expectancy (QALYs)	11.32	11.16	+0.17
Direct costs (EUR)	11,489.81	11,137.36	+352.45
Direct plus indirect costs (EUR)	12,322.91	12,924.88	−601.97
ICER from a healthcare payer perspective	EUR 2,134.36 per QALY gained		
ICER from a societal perspective	Onco*type* DX is dominant to standard care (cost and life saving)		

All costs are expressed in 2013 Euros (EUR). QALY, quality-adjusted life year; ICER, incremental cost-effectiveness ratio.

Evaluation of costs from a healthcare payer perspective showed that the use of Onco*type* DX was associated with an increase in mean costs of approximately EUR 352 per patient relative to standard care (Table 4). The acquisition costs of the test (EUR 3,180) were offset by substantial reductions in chemotherapy costs (EUR 1,508 due to chemotherapy sparing in low risk patients) and lower costs associated with distant recurrence (EUR 1,319 due to recurrence events avoided). Cost-effectiveness, expressed as an incremental cost-effectiveness ratio (ICER) of incremental costs divided by incremental effectiveness (QALYs), showed that Onco*type* DX is likely to be considered highly cost-effective from a healthcare payer perspective with an ICER of approximately EUR 2,134 per QALY gained versus standard care.

When costs were evaluated from a societal perspective, using Onco*type* DX to guide chemotherapy decision making was found to be cost saving versus standard care (Table 4). Inclusion of lost productivity costs in the modeling analysis meant that costs for eligible patients undergoing Onco*type* DX testing were on average EUR 602 lower than costs for those receiving standard care. This was primarily due to days off work associated with chemotherapy and management of side effects. As Onco*type* DX was both cost and life saving from a societal perspective, Onco*type* DX was considered to be dominant to standard care and no ICER was calculated based on societal costs.

**Fig 1:**
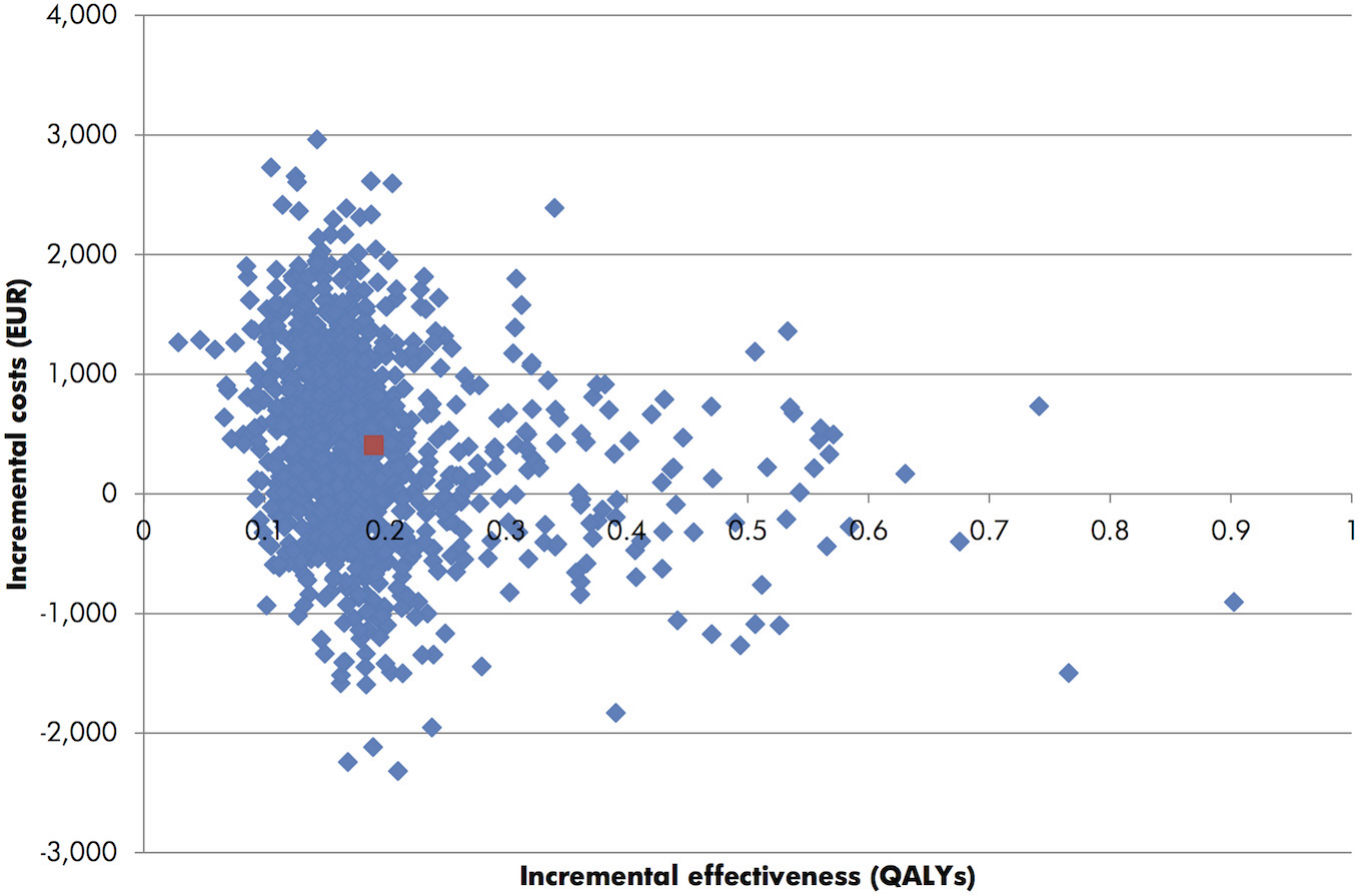
Cost-effectiveness scatterplot of the probabilistic sensitivity analysis. The cost-effectiveness scatterplot shows incremental costs (€) versus incremental effectiveness expressed in quality-adjusted life years (QALYs) for the comparison of Oncotype DX with standard care. Each blue point represents one iteration of the probabilistic sensitivity analysis (with data based on sampling from distributions around clinical and cost parameters). The red point indicates the mean (of 1,000 iterations).

Probabilistic sensitivity analysis with sampling from distributions around key model input with 1,000 iterations (Fig 1) showed that there was 30% probability that Onco*type* DX would be dominant to standard care (cost and life saving). Assuming a willingness to pay of EUR 30,000 per QALY gained, all 1,000 iterations indicated that Onco*type* DX would be cost-effective relative to standard care in the French private hospital setting.

One-way sensitivity analysis showed that Onco*type* DX remained cost-effective (or dominant) versus standard care with variation in a broad range of base case input parameters (Table 5). Decreasing the time horizon to 10 years increased the ICER for Onco*type* DX versus standard care to EUR 14,772.57 per QALY gained as the long-term benefits (in terms of recurrence events avoided) were not fully captured at this time horizon. Varying the costs and risk of recurrence also had a notable impact on the ICER. When upper 95% confidence limit of recurrence costs was used, Onco*type* DX became dominant to standard care. The same was true when the bounds of the upper 95% confidence intervals for the relative risks of recurrence were used in the analysis. Other one-way sensitivity analysis had a limited effect on the cost-effectiveness of Onco*type* DX versus standard care.

**Table 5:** Summary of one-way sensitivity analysis outcomes for Onco*type* DX testing versus standard care.

	Quality-adjusted life expectancy (QALYs)			Direct costs (EUR)			ICER*/Outcomes
	Onco*type* DX	Standard care	Difference	Onco*type* DX	Standard care	Difference	
Base case	11.32	11.16	0.17	11,489.81	11,137.36	+352.45	2,134.36
Time horizon 10 years	6.27	6.22	0.05	8,407.83	7,677.39	+730.44	14,772.57
Time horizon 20 years	9.79	9.67	0.12	10,589.40	10,171.99	+417.41	3,476.99
Time horizon 40 years	11.58	11.41	0.17	11,636.39	11,287.70	+348.69	2,003.20
Discount rate 0%	17.44	17.14	0.30	15,228.66	15,355.73	-127.07	DOMINANT
Discount rate 8%	8.12	8.02	0.10	9,509.94	8,870.69	+639.25	6,268.62
Chemotherapy costs UL	11.32	11.16	0.17	11,617.57	11,411.48	+206.09	1,248.04
Chemotherapy costs LL	11.32	11.16	0.17	11,362.01	10,863.14	+498.87	3,021.02
Recurrence costs UL	11.32	11.16	0.17	29,210.95	32,201.15	-2,990.20	DOMINANT
Recurrence costs LL	11.32	11.16	0.17	7,999.83	6,989.08	+1,010.75	6,120.87
Relative risk of recurrence UL	11.44	11.24	0.21	10,285.62	10,352.64	-67.02	DOMINANT
Relative risk of recurrence LL	11.21	11.09	0.13	12,537.98	11,820.41	+717.57	5,651.85
Net change in chemotherapy use UL in low RS group	11.32	11.16	0.17	11,376.21	11,137.36	+238.84	1,435.46
Net change in chemotherapy use LL in low RS group	11.32	11.16	0.16	11,836.95	11,137.36	+699.59	4,337.26
Net change in chemotherapy use UL in intermediate RS group	11.32	11.16	0.17	11,371.16	11,137.36	+233.79	1,404.67
Net change in chemotherapy use LL in intermediate RS group	11.32	11.16	0.16	11,607.84	11,137.36	+470.48	2,871.70
Net change in chemotherapy use UL in high risk group	11.36	11.16	0.20	11,248.41	11,137.36	+111.04	551.70
Net change in chemotherapy use LL in high risk group	11.29	11.16	0.13	11,731.22	11,137.36	+593.86	4,604.02
10 year risk of recurrence UL	11.27	11.08	0.19	11,928.15	11,740.32	+187.83	992.25
10-year risk of recurrence LL	11.37	11.23	0.14	11,030.52	10,489.27	+541.45	3,872.44
Survival post recurrence UL	11.20	11.03	0.17	12,720.28	12,367.83	+352.45	2,134.54
Survival post recurrence LL	11.45	11.29	0.17	10,187.78	9,835.33	+352.45	2,134.18

QALY, quality-adjusted life year; EUR, 2013 Euros; ICER, incremental cost-effectiveness ratio

* ICERs are presented in EUR per QALY gained; RS, Recurrence Score.

## Discussion

The present study is the first multicenter analysis to report the cost of chemotherapy in patients with early-stage breast cancer treated in private hospitals in France. Seven hospitals from different cities were involved, taking into account differences in management of adjuvant chemotherapy, and all resources related to the management of toxicity, including both prophylactic and symptomatic prescriptions, were accounted for with a high degree of detail. These data are of significant importance, not only because 43% of surgeries for early stage breast cancer are performed at private hospitals, but because the complexity of the reimbursement landscape in France means that health economic analysis will be key in optimizing reimbursement decision in the months and years ahead for both public and private payers. The costing analysis reported in this paper presents a valuable resource for researchers investigating reimbursement issues for patients with early-stage breast cancer. It showed that prophylactic prescriptions and chemotherapy administration costs, along with absenteeism, were the biggest contributors to total costs from both the healthcare payer and societal perspectives.

The utility of these cost estimates was demonstrated in the present cost-effectiveness analysis of using the Onco*type* DX recurrence score to guide chemotherapy decision making versus standard care. Based on an international meta-analysis and a previously published model, the modeling study showed that Onco*type* DX is likely to improve both life expectancy and quality-adjusted life expectancy relative to standard care in eligible patients with early-stage breast cancer. Moreover, the acquisition costs of the test were largely offset by reductions in chemotherapy costs (chemotherapy sparing) and reductions in distant recurrence. As a result, Onco*type* DX is found to be dominant to standard care from a societal perspective and should be considered highly cost-effective by commonly quoted standards from a healthcare payer perspective relative to standard care. The findings of the cost-effectiveness evaluation were consistent with those of previous analyses on Onco*type* DX, which have shown that Onco*type* DX was also likely to be dominant to standard care (cost and life saving) in the public hospital setting from a societal perspective.[22] Comparable findings in the public and private sectors in France support the generalizability of the present study and are consistent with recent observations that no notable differences exist in the management of breast cancer between public and private centers in France.[34] Moreover, a recent systematic review identified 18 published cost-effectiveness analyses of Onco*type* DX, across a range of country-settings, which consistently showed that Onco*type* DX is associated with improved clinical outcomes and is either cost-effective or cost saving relative to standard care in women with ER+, HER2−, early-stage breast cancer.

The evaluation of costs was limited to those associated with adjuvant chemotherapy. The objective was not to provide an estimate of the total costs of breast cancer care, but rather to focus on the additional costs of adjuvant chemotherapy, and as a result costs associated with radiotherapy, endocrine therapy, and long-term toxicities were not included. In 2012, Laas *et al*. published an evaluation of the costs of adjuvant chemotherapy in France from the societal perspective and reported an estimate of EUR 15,740 per patient.[7] This estimate is higher than that reported here (EUR 10,305). However, in the present analysis, all chemotherapy drugs were included in the unique T2A tariff paid to the hospital (no drugs were paid in addition) and, in 2011, agents such as docetaxel were not included in the T2A tariff (but were paid in addition to the T2A tariff). Moreover, there is a notable difference in the mean T2A payments for public (EUR 390.28 in 2011) and private hospitals (EUR 297.48 in 2013) due to the two reimbursement regimens implemented by health authorities, which is likely also to contribute to the difference in total cost estimates in the two studies.

Long-term modeling analyses are inevitably associated with limitations, as assumptions are invariably required to make long-term estimates of costs and clinical outcomes. In the present analysis, a previously published health economic model was used to minimize the potential impact of unvalidated assumptions and model (structural) uncertainty. A potential short-coming of the modeling analysis is that it used decision impact data (on how Onco*type* DX influences decision on adjuvant chemotherapy) from an international meta-analysis. In the absence of country-specific decision impact data, one-way sensitivity analyses were performed that showed altering the decision for patients with a low, intermediate or high risk of recurrence based on RS within plausible ranges, did not alter the conclusions of the analysis (Onco*type* DX remained cost-effective or cost saving). Further, even though the model captured more than short-term adverse effects of chemotherapy treatment, it did not take into account long-term adverse-events such as abnormal heart function or impaired cognitive function. This limitation is conservative, as the chemotherapy sparing benefits associated with Onco*type* DX are not fully captured within the present modeling framework. Similarly, a conservative assumption was made in that no utility increment associated with the use of the Onco*type* DX test was captured in the analysis. It has been shown that the use of the Onco*type* DX breast cancer assay increases patients and physicians confidence in their decision and decreases anxiety in patients.[35] It would therefore be reasonable to assume that the value of the information delivered by gene expression tests such as Onco*type* DX should translate into quality of life benefits. In addition, the cost of recurrence was estimated from a healthcare payer perspective and may, therefore, represent an underestimate in the societal analysis.[29] Although the clinical validity of the Onco*type* DX test has been evaluated retrospectively in several studies involving over 3,300 patients, prospective clinical data (level 1 evidence) are not yet available, though three prospective trial are currently ongoing. The findings from these studies may offer a more robust evidence base not only for the test itself, but also for future health economic evaluations.

In addition to the retrospective costing analysis and cost-effectiveness evaluation, an analysis of revenue from the perspective of private hospitals was performed to help put the present findings in context for healthcare payers. The analysis was deigned to estimate marginal revenue, defined as the revenue generated by one patient with adjuvant chemotherapy, for scenarios with and without the use of the Onco*type* DX test based on French data describing patient characteristics and the meta-analysis of Onco*type* DX decision impact studies.[4, 13] The analysis showed that, based on an average net profit margin of 2.9% reported for Générale de Santé private hospitals, there would be an estimated revenue loss for private hospitals of at least EUR 3,203 per patient tested as the test is not currently reimbursed.[36] This evaluation did not integrate the potential benefits associated with improved hospital reputation and enhanced attractiveness for patients of using Onco*type* DX (due to improved clinical outcomes). Moreover, offering the test is likely to free up resources (e.g. medical personnel, chairs for intravenous infusion, etc.), which could be allocated for the patients who still undergo chemotherapy with higher recurrence scores.

Onco*type* DX was chosen for the present analysis as it is supported by a greater evidence base than other gene expression profiling or expanded immunohistochemistry tests.[37] However, several other tests exist that may support improved chemotherapy decision making and beneficial economic outcomes. Although meaningful comparisons of cost-effectiveness between tests are not currently possible in the absence of data from head-to-head decision impact studies, Ward *et al*. (2013) published a systematic review of clinical and cost-effectiveness of current prognostic tools in guiding the use of adjuvant chemotherapy in patients with early breast cancer performed to support the development of guidelines for the National Institute of Health and Care Excellence (NICE) in the UK.[38] A cost-effectiveness evaluation was performed for Onco*type* DX, IHC4, MammaPrint and Mammostrat tests in comparison with standard clinical practice in England and Wales (as evidence for five other tests was too limited: PAM50, NPI+, BCI, BluePrint and Randox). The authors concluded that the clinical evidence base for Onco*type* DX was the most robust (and was associated with an ICER GBP 26,940 per QALY gained versus standard care). The authors also noted that treatment guided using IHC4 has the most potential to be cost-effective but that the evidence base to support IHC4 “needs significant further research.”

As highlighted in the 2013 St. Gallen guidelines, there is discord between the reimbursement status in several European countries, including France.[21] Following the French Plan Hôpital reforms in 2007, the government required hospitals to adopt an activity-based Disease Related Groups (DRG) pricing system: the tarification à l'activité (T2A). The approach is based on Groupes Homogènes de Séjour (GHS or uniform hospitalization groups). Hospitals receive a fixed price for treating a patient allocated to a GHS. The GHS price includes all medicine and resources used to treat the patient. However, innovative treatments often do not fall within existing T2A definitions, particularly if the act does not resemble a previously established practice, as can be the case for gene expression profiling. Creating a new T2A takes 2–3 years in the best case. Intermediate funding opportunities exist through regional authorities or project tenders, but these are limited. As a result, the launch of a new product can therefore be directly affected by the ability to obtain funding outside of the T2A system. If no funds are available, public and private hospitals will be reluctant to use the product or may simply be unable to do so. In 2014, the 21 gene assay was funded through a very limited number of hospitals and regional authorities (Agence Régionale de Santé).

The absence of a national funding mechanism allowing the controlled used of innovative tests, such as Onco*type* DX, has therefore created a situation of unequal patient access. Similar situations exist in a number of other countries, and this situation raises the question of whether traditional models of reimbursement are adequate to deal with issues such as genetic testing in the public health sphere in the era of personalized medicine.[39, 40] Since 2013, French health reform had a clear focus on patient access to personalized medicine (passed by the French Congress in April 2015), but currently the reimbursement of prognostic tests remain unclear.[41] As reimbursement remains the largest hurdle to the wider adoption of personalized medicine products, alternative payment methods are emerging. Public-private partnerships, such as AstraZeneca-funded testing in non-small cell lung cancer patients to identify those who would respond to its gefitinib, may offer a potential solution for some conditions. Patient advocacy groups may also play a role; for example the Cystic Fibrosis Foundation’s Mutation Analysis Program offered genetic testing to identify those who would benefit from benefit from an orphan drug (ivacaftor). Direct patient payments represent another option, but with the obvious caveat of inequality. The ethical committee from the Ligue Nationale contre le Cancer, one of the leading patient cancer associations in France, underlined that the absence of coverage presents a dilemma where only wealthy patients will be able to afford the test.[42] This report indicates that some oncologists prefer to dissimulate the test availability to patients that may benefit from it, but simply cannot afford it. During the period where the test is not reimbursed, oncologists are still confronted with their obligation of means with respect to all patients. Prescribing chemotherapies to resistant patients that could have been detected with the test can present medico-legal risks as patients could claim a loss of chance, resulting in litigation involving both physicians and health care institutions. Moreover, it creates the potential for unequal access by socioeconomic status, with more affluent patients opting to pay privately for testing, an option that may be beyond the means of other individuals. From an economic perspective, the present T2A French system reimburses adjuvant chemotherapies in all cases, even for resistant patients. To maximize revenues, public and private hospitals are incentivized not to use gene expression profiling or expanded immunohistochemistry tests and continue prescribing chemotherapies even when unnecessary, which represents a clinical, ethical and economic impasse.

## Conclusion

Our study provides evidence of the cost-effectiveness of Onco*type* DX from a healthcare payer perspective as well as the cost savings from a societal perspective. However, the analysis also raises the questions around reimbursement and access to the test for patients and healthcare professionals, as it is not covered by primary payers such as the social security. The delay in coverage of molecular testing has the potential to erode the quality of the French healthcare system. Budget constraints are likely to mean that tests like Onco*type* DX will remain underused in France, thus depriving patients of technologies that could improve clinical outcomes and allow healthcare professionals to better allocate hospital resources to improve the standard of care for all patients.

## Supporting Information

S1 File Patient characteristics, protocols and cost-effectiveness results for Onco*type* DX per cycle. Table B1. Distribution of sick leave for working patients. Table B2. ART-Patients characteristics. Table B3. Average dosage of chemotherapy drug per cycle in mg/m² per protocol. Table B4. Patient characteristics by hospital. Table B5. Distribution of side effects per cycle. Table B6. Home care. Table B7. Hospitalization characteristics. Table B8. Income for hospital per cycle. Table B9. Income for hospital by patient. Table B10. Aggregated costs per patient–Insurance perspective. Table B11. Aggregated costs per cycle–Insurance perspective. Table B12. Monitoring–Distribution of cycle with laboratory tests. Table B13. Monitoring–Consultation per cycle. Table B14. Pre-chemotherapy tests. Table B15. Protocols–Number of cycles. Table B16. Average cost by patient–Societal perspective. Table B17. Average cost per cycle–Societal perspective. Table B18. Distribution of chemotherapy strategies and protocols. Table B19. Distribution of cycle and reason for symptomatic prescription. Table B20. Transportation and distribution of transport.
(RTF)
