# Supplementary material for: Economic Impact of Gene Expression Profiling in Patients with Early-Stage Breast Cancer in France
Source: PLoS One. 2015 Jun 18;10(6):e0128880. doi: 10.1371/journal.pone.0128880 (PMC4472722; doi:10.1371/journal.pone.0128880)
Supplement: S1 File — Table B1. Distribution of sick leave for working patients. Table B2. ART-Patients characteristics. Table B3. Average dosage of chemotherapy drug per cycle in mg/m² per protocol. Table B4. Patient characteristics by hospital. Table B5. Distribution of side effects per cycle. Table B6. Home care. Table B7. Hospitalization characteristics. Table B8. Income for hospital per cycle. Table B9. Income for hospital by patient. Table B10. Aggregated costs per patient–Insurance perspective. Table B11. Aggregated costs per cycle–Insurance perspective. Table B12. Monitoring–Distribution of cycle with laboratory tests. Table B13. Monitoring–Consultation per cycle. Table B14. Pre-chemotherapy tests. Table B15. Protocols–Number of cycles. Table B16. Average cost by patient–Societal perspective. Table B17. Average cost per cycle–Societal perspective. Table B18. Distribution of chemotherapy strategies and protocols. Table B19. Distribution of cycle and reason for symptomatic prescription. Table B20. Transportation and distribution of transport. (RTF) [file pone.0128880.s001.rtf]

Economic impact of gene expression profiling in patients with early-stage breast cancer in France
Supporting Information File (S1)

Gregory Katz*1,9, Olivier Romano2,10, Cyril Foa3, Anne-Lise Vataire4,11, Jean-Victor Chantelard5, Robert Hervé3, Hugues Barletta6, Axel Durieux7, Jean-Pierre Martin8, Rémy Salmon7

Affiliations:	1  ESSEC Business School, Chair of Therapeutic Innovation, Paris-Singapore
2  Générale de Santé, Hôpital Privé la Louvière, Lille, France
3  Générale de Santé, Hôpital Privé Clairval, Marseille, France
4  Creativ-Ceutical, Paris, France
5  Générale de Santé, Hôpital Privé Paul d'Egine, Champigny-sur-Marne, France
6  Générale de Santé, Hôpital Privé Drôme-Ardèche, Guilherand-Granges, France
7  Générale de Santé, Hôpital Privé des Peupliers, Paris, France
8  Générale de Santé, Hôpital Privé Jean Mermoz, Lyon, France
9  Fondation Générale de Santé, Paris, France
10  Générale de Santé, Hôpital Privé Villeneuve-d'Ascq, Villeneuve-d'Ascq, France
11 Université Lyon Claude Bernard, Lyon, France


Table B1. Distribution of sick leave for working patients

		Pôle Lille	Paul D'Egine	Clairval	Drôme Ardèche	Peupliers	Jean Mermoz		total	
										
Working patients	Missing	6 (24.00%)	0	0	0	0	0	6 (	5.66%)	
										
	Valid values	19	17	16	21	9	18		100	
									
	Yes	14 (73.68%)	11 (64.71%)	11 (68.75%)	9 (42.86%)	8 (88.89%)	12 (66.67%)	65 (65.00%)	
									
	No	5 (26.32%)	6 (35.29%)	5 (31.25%)	12 (57.14%)	1 (11.11%)	6 (33.33%)	35 (35.00%)	
										
Sick leave for	Missing	0	0	0	0	0	0		0	
working patients										
										
	Valid values	14	11	11	9	8	12		65	
									
	Yes	9 (64.29%)	11 (100.00%)	11 (100.00%)	9 (100.00%)	8 (100.00%)	11 (91.67%)	59 (90.77%)	
										
	No	5 (35.71%)	0	0	0	0	1 (8.33%)	6 (	9.23%)	
										


Table B2. Patients characteristics
Patient characteristics	Total (N=106)	
			
Age (year)	Mean (Sd)	53.2 (11.3)	
			
Height (cm)	Mean (Sd)	161.4 (7.4)	
			
Weight (kg)	Mean (Sd)	64.9 (13.4)	
			
Surface (m²)	Mean (Sd)	1.7 (0.2)	
			
Profession (%)		n=92	
	Agriculture: primary sector	3.2%	
	Higher intellectual	16.3%	
	Intermediate occupation	7.6%	
	Employee	33.7%	
	Labourer	1.1%	
	Retired	27.1%	
	Without activity	10.8%	
			
Stage (%)		n=100	
	1	50.0%	
	2	50.0%	
			
pTNM (%)		n=106	
	T1n0	49.0%	
	T2n0	47.1%	
	T3n0	3.7%	
			
HER2 (%)		n=106	
	-VE	100.0%	
	+VE	0%	
			
RE (%)		n=106	
	-VE	100.00%	
	+VE	0%	
			
RP (%)		n=106	
	-VE	16.9%	
	+VE	83.1%	

Table B3. Average dosage of chemotherapy drug per cycle in mg/m² per protocol

										
		Pôle Lille	Paul D'Egine	Clairval	Drôme Ardèche	Peupliers	Jean Mermoz	Total	Test	
										
DOCET	Missing	0	-	0	0	0	0	0	W = 24.70	
AXEL										
										
	Nb of valid	73	0	15	33	22	16	159	p < 0.0001	
	values									
										
	Mean (Sd)	165.07 (18.87)	-	147.87 (25.57)	170.61 (16.38)	148.64 (21.45)	153.13 (7.93)	161.12 (20.31)		
										
	Min-Max	[120.0;200.0]	-	[111.0;181.0]	[140.0;200.0]	[100.0;170.0]	[140.0;160.0]	[100.0;200.0]		
										
DOCET	Missing	-	-	-	0	0	0	0	T = 5.915	
AXEL										
										
	Nb of valid	0	0	0	26	4	48	78	p = 0.004	
	values									
										
	Mean (Sd)	-	-	-	125.38 (11.74)	100.00 (0.00)	124.17 (15.48)	123.33 (14.87)		
										
	Min-Max	-	-	-	[110.0;150.0]	[100.0;100.0]	[90.0;140.0]	[90.0;150.0]		
										
CYCLO	Missing	-	-	-	0	0	0	0	T = 56.927	
PHOSP										
HAMID										
										
	Nb of valid	0	0	0	26	4	48	78	p < 0.0001	
	values									
										
	Mean (Sd)	-	-	-	1002.31	1500.00 (0.00)	944.79	992.44		
					(99.21)		(103.78)	(156.85)		
										
	Min-Max	-	-	-	[850.0;	[1500.0;	[750.0;	[750.0;		
					1190.0]	1500.0]	1100.0]	1500.0]		
										
DOCETAXEL	Missing	-	0	-	-	-	-	0	W = NA	
										
										
	Nb of valid	0	18	0	0	0	0	18	p = NA	
	values									
										
	Mean (Sd)	-	106.67 (10.15)	-	-	-	-	106.67 (10.15)		
										
	Min-Max	-	[90.0;125.0]	-	-	-	-	[90.0;125.0]		
										
EPIRUBICINE	Missing	-	0	-	-	-	-	0	W = NA	
										
										
	Nb of valid	0	18	0	0	0	0	18	p = NA	
	values									
										
	Mean (Sd)	-	106.67 (10.15)	-	-	-	-	106.67 (10.15)		
										
	Min-Max	-	[90.0;125.0]	-	-	-	-	[90.0;125.0]		
										
CYCLOPHOSPHAMID	Missing	-	0	-	-	-	-	0	W = NA	
										
										
										
	Nb of valid	0	18	0	0	0	0	18	p = NA	
	values									
										
	Mean (Sd)	-	721.67	-	-	-	-	721.67		
			(104.04)					(104.04)		
										
	Min-Max	-	[500.0;850.0]	-	-	-	-	[500.0;850.0]		
										
DOXORUBICINE	Missing	-	-	0	-	-	-	0	W = NA	
										
										
										
	Nb of valid	0	0	15	0	0	0	15	p = NA	
	values									
										
	Mean (Sd)	-	-	106.60 (8.95)	-	-	-	106.60 (8.95)		
										
	Min-Max	-	-	[97.0;120.0]	-	-	-	[97.0;120.0]		
										
CYCLOPHOSPHAMID	Missing	-	-	0	-	-	-	0	W = NA	
										
										
										
	Nb of valid	0	0	15	0	0	0	15	p = NA	
	values									
	Mean (Sd)	-	-	1065.20	-	-	-	1065.20		
				(89.85)				(89.85)		
										
	Min-Max	-	-	[966.0;	-	-	-	[966.0;		
				1200.0]				1200.0]		
										
EPIRUBICINE	Missing	0	-	-	-	-	-	0	W = NA	
										
										
	Nb of valid	1	0	0	0	0	0	1	p = NA	
	values									
										
	Mean (Sd)	170.00 (--)	-	-	-	-	-	170.00 (--)		
										
	Min-Max	[170.0;170.0]	-	-	-	-	-	[170.0;170.0]		
										
EPIRUBICINE	Missing	-	-	0	-	0	-	0	T = 1.554	
										
										
	Nb of valid	0	0	18	0	1	0	19	p = 0.23	
	values									
										
	Mean (Sd)	-	-	169.11 (22.73)	-	140.00 (--)	-	167.58 (23.08)		
										
	Min-Max	-	-	[148.0;200.0]	-	[140.0;140.0]	-	[140.0;200.0]		
										
CYCLOPHOSPHAMID	Missing	-	-	0	-	0	-	0	T = 0.67	
										
										
										
	Nb of valid	0	0	18	0	1	0	19	p = 0.42	
	values									
										
	Mean (Sd)	-	-	1014.67	-	900.00 (--)	-	1008.63		
				(136.37)				(135.11)		
										
	Min-Max	-	-	[888.0;	-	[900.0;900.0]	-	[888.0;		
				1200.0]				1200.0]		
										
EPIRUBICINE	Missing	-	-	-	-	-	0	0	W = NA	
										
										
	Nb of valid	0	0	0	0	0	1	1	p = NA	
	values									
										
	Mean (Sd)	-	-	-	-	-	150.00 (--)	150.00 (--)		
										
	Min-Max	-	-	-	-	-	[150.0;150.0]	[150.0;150.0]		
										
FLUOROURACIL	Missing	-	-	-	-	-	0	0	W = NA	
										
										
										
	Nb of valid	0	0	0	0	0	1	1	p = NA	
	values									
										
	Mean (Sd)	-	-	-	-	-	750.00 (--)	750.00 (--)		
										
	Min-Max	-	-	-	-	-	[750.0;750.0]	[750.0;750.0]		
										
EPIRUBICINE	Missing	0	0	0	-	-	0	0	W = 61.72	
										
										
	Nb of valid	76	78	28	0	0	17	199	p < 0.0001	
	values									
										
	Mean (Sd)	173.16 (13.68)	151.41 (15.31)	154.00 (24.89)	-	-	157.06 (7.72)	160.56 (18.77)		
										
	Min-Max	[140.0;200.0]	[110.0;170.0]	[113.0;187.0]	-	-	[150.0;170.0]	[110.0;200.0]		
										
CYCLOPHOSPHAMID	Missing	0	0	0	-	-	0	0	W = 61.84	
										
										
										
	Nb of valid	76	78	28	0	0	17	199	p < 0.0001	
	values									
										
	Mean (Sd)	863.29 (66.92)	764.36 (69.82)	792.96	-	-	770.59 (25.36)	806.70 (86.72)		
				(114.97)						
										
	Min-Max	[700.0;	[570.0;870.0]	[568.0;935.0]	-	-	[750.0;800.0]	[568.0;		
		1000.0]						1000.0]		
										
FLUOROURACIL	Missing	0	0	0	-	-	0	0	W = 61.84	
										
										
										
	Nb of valid	76	78	28	0	0	17	199	p < 0.0001	
	values									
										
	Mean (Sd)	863.29 (66.92)	764.36 (69.82)	792.96	-	-	770.59 (25.36)	806.70 (86.72)		
				(114.97)						
										
	Min-Max	[700.0;	[570.0;870.0]	[568.0;935.0]	-	-	[750.0;800.0]	[568.0;		
		1000.0]						1000.0]		
										
PACLITAXEL	Missing	-	-	-	0	-	0	0	T = 0.533	
										
										
	Nb of valid	0	0	0	8	0	2	10	p = 0.49	
	values									
										
	Mean (Sd)	-	-	-	125.00 (9.26)	-	130.00 (0.00)	126.00 (8.43)		
										
	Min-Max	-	-	-	[120.0;140.0]	-	[130.0;130.0]	[120.0;140.0]		
										
PACLITAXEL	Missing	-	-	-	0	-	-	0	W = NA	
										
										
	Nb of valid	0	0	0	2	0	0	2	p = NA	
	values									
										
	Mean (Sd)	-	-	-	330.00 (0.00)	-	-	330.00 (0.00)		
										
	Min-Max	-	-	-	[330.0;330.0]	-	-	[330.0;330.0]		
										
CYCLOPHOSPHAMID	Missing	-	-	-	0	-	-	0	W = NA	
										
										
										
	Nb of valid	0	0	0	2	0	0	2	p = NA	
	values									
										
	Mean (Sd)	-	-	-	850.00 (0.00)	-	-	850.00 (0.00)		
										
	Min-Max	-	-	-	[850.0;850.0]	-	-	[850.0;850.0]		
										


Table B4. Patient characteristics by hospital

					Drôme		Jean			
		Pôle Lille	Paul D'Egine	Clairval	Ardèche	Peupliers	Mermoz	Total	test	
										
Age	Missing	0	0	0	0	0	0	0	T = 1.058	
										
	Nb of valid	25	17	16	21	9	18	106	p = 0.39	
	values									
										
	Mean (Sd)	52.48 (11.34)	56.41 (10.24)	52.00 (13.55)	56.29 (10.27)	48.89 (12.60)	50.89 (10.46)	53.22 (11.31)		
										
	95% CI	[47.80;	[51.15;	[44.78;	[51.61;	[39.20;	[45.69;	[51.04;		
		57.16]	61.67]	59.22]	60.96]	58.58]	56.09]	55.40]		
										
	Min-Max	[29.0;73.0]	[41.0;72.0]	[35.0;81.0]	[35.0;75.0]	[36.0;67.0]	[34.0;68.0]	[29.0;81.0]		
										
	Median	52.0	59.0	50.5	56.0	47.0	50.0	52.0		
										
	Q1-Q3	[46.0;61.0]	[47.0;64.0]	[42.0;57.5]	[50.0;65.0]	[37.0;59.0]	[41.0;60.0]	[46.0;62.0]		
										
Height (cm)	Missing	0	0	0	0	0	0	0	T = 1.487	
										
	Nb of valid	25	17	16	21	9	18	106	p = 0.20	
	values									
										
	Mean (Sd)	164.12 (7.92)	158.94 (5.79)	163.00 (6.76)	160.05 (5.51)	162.00	160.11 (8.39)	161.45 (7.42)		
						(10.28)				
										
	95% CI	[160.85;	[155.96;	[159.40;	[157.54;	[154.10;	[155.94;	[160.02;		
		167.39]	161.92]	166.60]	162.56]	169.90]	164.28]	162.88]		
										
	Min-Max	[153.0;	[152.0;	[152.0;	[150.0;	[151.0;	[142.0;	[142.0;		
		190.0]	170.0]	178.0]	169.0]	182.0]	175.0]	190.0]		
										
	Median	165.0	158.0	162.0	160.0	163.0	160.0	160.0		
										
	Q1-Q3	[160.0;	[154.0;	[159.0;	[155.0;	[153.0;	[158.0;	[156.0;		
		168.0]	162.0]	167.5]	165.0]	165.0]	164.0]	165.0]		
										
Weight (kg)	Missing	0	0	0	0	0	0	0	T = 0.495	
										
	Nb of valid	25	17	16	21	9	18	106	p = 0.78	
	values									
										
	Mean (Sd)	67.32 (10.98)	62.29 (10.25)	63.81 (13.15)	67.00 (15.45)	64.33 (20.29)	62.83 (13.49)	64.91 (13.37)		
										
	95% CI	[62.79;	[57.03;	[56.80;	[59.97;	[48.74;	[56.12;	[62.33;		
		71.85]		67.56]		70.82]	74.03]	79.93]	69.54]		67.48]		
										
	Min-Max	[51.0;92.0]	[46.0;85.0]	[50.0;90.0]	[47.0;100.0]	[46.0;110.0]	[40.0;85.0]	[40.0;110.0]		
													
	Median	65.0		61.0		56.5	64.0	59.0	58.5		62.0		
										
	Q1-Q3	[60.0;76.0]	[55.0;66.0]	[55.0;71.5]	[57.0;72.0]	[51.0;70.0]	[53.0;72.0]	[55.0;73.0]		
													
Surface (m²)	Missing	0		0		0	0	0	0		0	T = 0.694	
													
	Nb of valid	25		17		16	21	9	18		106	p = 0.63	
	values												
										
	Mean (Sd)	1.75 (0.15)	1.65 (0.14)	1.69 (0.20)	1.72 (0.20)	1.69 (0.30)	1.66 (0.19)	1.70 (0.19)		
										
	95% CI	[1.68;1.81]	[1.58;1.73]	[1.59;1.80]	[1.62;1.81]	[1.46;1.92]	[1.57;1.75]	[1.66;1.73]		
										
	Min-Max	[1.5;2.1]	[1.4;1.9]	[1.5;2.1]	[1.4;2.1]	[1.4;2.3]	[1.3;1.9]	[1.3;2.3]		
													
	Median	1.7		1.7		1.6	1.7	1.6	1.6		1.7		
										
	Q1-Q3	[1.7;1.8]	[1.5;1.7]	[1.6;1.8]	[1.6;1.8]	[1.5;1.8]	[1.5;1.8]	[1.6;1.8]		
											
Profession[n(	Missing	7 (28.00%)	1 (	5.88%)	3 (18.75%)	1 (4.76%)	2 (22.22%)	0	14 (13.21%)	X = 33.53	
%)]													
													
	Valid values	18		16		13	20	7	18		92	p = 0.30	
													
	Agriculture:	0	1 (	6.25%)	1 (	7.69%)	0	0	1 (5.56%)	3 (	3.26%)		
	primary sector												
										
	Higher	2 (11.11%)	3 (18.75%)	2 (15.38%)	0	3 (42.86%)	5 (27.78%)	15 (16.30%)		
	intellectual												
												
	Intermediate	1 (5.56%)		0	2 (15.38%)	2 (10.00%)	0	2 (11.11%)	7 (	7.61%)		
	occupation												
										
	Employee	10 (55.56%)	6 (37.50%)	2 (15.38%)	6 (30.00%)	3 (42.86%)	4 (22.22%)	31 (33.70%)		
													
	Labourer	0		0	1 (	7.69%)	0	0	0	1 (	1.09%)		
										
	Retired	5 (27.78%)	5 (31.25%)	3 (23.08%)	7 (35.00%)	1 (14.29%)	4 (22.22%)	25 (27.17%)		
													
	Without activity	0	1 (6.25%)	2 (15.38%)	5 (25.00%)	0	2 (11.11%)	10 (10.87%)		
											
Stade[n(%)]	Missing	0	0	0	0	1 (11.11%)	5 (27.78%)	6 (	5.66%)	X = 5.65	
											
	Valid values	25	17	16	21	8	13		100	p = 0.34	
										
	1	15 (60.00%)	10 (58.82%)	4 (25.00%)	11 (52.38%)	4 (50.00%)	6 (46.15%)	50 (50.00%)		
										
	2	10 (40.00%)	7 (41.18%)	12 (75.00%)	10 (47.62%)	4 (50.00%)	7 (53.85%)	50 (50.00%)		
											
pTNM[n(%)]	Missing	0	0	0	0	0	0		0	X = 11.36	
											
	Valid values	25	17	16	21	9	18		106	p = 0.33	
										
	T1n0	14 (56.00%)	11 (64.71%)	6 (37.50%)	11 (52.38%)	2 (22.22%)	8 (44.44%)	52 (49.06%)		
										
	T2n0	11 (44.00%)	6 (35.29%)	8 (50.00%)	10 (47.62%)	6 (66.67%)	9 (50.00%)	50 (47.17%)		
											
	T3n0	0	0	2 (12.50%)	0	1 (11.11%)	1 (5.56%)	4 (	3.77%)		
											
HER2[n(%)]	Missing	0	0	0	0	0	0		0	X = NA	
											
	Valid values	25	17	16	21	9	18		106	p = NA	
											
	-VE	25 (100.00%)	17 (100.00%)	16 (100.00%)	21 (100.00%)	9 (100.00%)	18 (100.00%)		106		
								(100.00%)		
											
RE[n(%)]	Missing	0	0	0	0	0	0		0	X = NA	
											
	Valid values	25	17	16	21	9	18		106	p = NA	
											
	+VE	25 (100.00%)	17 (100.00%)	16 (100.00%)	21 (100.00%)	9 (100.00%)	18 (100.00%)		106		
								(100.00%)		
											
RP[n(%)]	Missing	0	0	0	0	0	0		0	X = 9.78	
											
	Valid values	25	17	16	21	9	18		106	p = 0.08	
										
	-VE	3 (12.00%)	0	2 (12.50%)	6 (28.57%)	1 (11.11%)	6 (33.33%)	18 (16.98%)		
										
	+VE	22 (88.00%)	17 (100.00%)	14 (87.50%)	15 (71.43%)	8 (88.89%)	12 (66.67%)	88 (83.02%)		
											

	

Table B5. Distribution of side effects per cycle

		Pôle Lille	Paul D'Egine	Clairval	Drôme Ardèche	Peupliers	Jean Mermoz		Total	test	
																		
Alopecia	Missing		0		0		0		0		0		0			0	X = 241.96	
													
	Yes	12 (	8.00%)	23 (22.55%)	67 (88.16%)	2 (	1.80%)	10 (18.52%)	12 (14.29%)	126	(21.84%)		
											
	No	138 (92.00%)	79 (77.45%)	9 (11.84%)	109 (98.20%)	44 (81.48%)	72 (85.71%)	451	(78.16%)		
																		
Anemia	Missing		0		0		0		0		0		0			0	X = 18.41	
																	
	Yes		0	8 (	7.84%)	3 (	3.95%)	2 (	1.80%)	2 (	3.70%)		0	15 (	2.60%)		
											
	No	150 (100.00%)	94 (92.16%)	73 (96.05%)	109 (98.20%)	52 (96.30%)	84 (100.00%)	562	(97.40%)		
																		
Asthenia	Missing		0		0		0		0		0		0			0	X = 75.65	
													
	Yes	48 (32.00%)	23 (22.55%)	9 (11.84%)	1 (	0.90%)	20 (37.04%)	1 (	1.19%)	102	(17.68%)		
											
	No	102 (68.00%)	79 (77.45%)	67 (88.16%)	110 (99.10%)	34 (62.96%)	83 (98.81%)	475	(82.32%)		
																		
Allergy	Missing		0		0		0		0		0		0			0	X = 10.47	
																		
	Yes		0	1 (	0.98%)		0	5 (	4.50%)	1 (	1.85%)	2 (	2.38%)	9 (		1.56%)		
											
	No	150 (100.00%)	101 (99.02%)	76 (100.00%)	106 (95.50%)	53 (98.15%)	82 (97.62%)	568	(98.44%)		
																		
Cardiotoxi	Missing		0		0		0		0		0		0			0	X = 5.58	
city																		
																		
	Yes	3 (	2.00%)	2 (	1.96%)	2 (	2.63%)		0		0		0	7 (		1.21%)		
											
	No	147 (98.00%)	100 (98.04%)	74 (97.37%)	111 (100.00%)	54 (100.00%)	84 (100.00%)	570	(98.79%)		
																		
Bone pain	Missing		0		0		0		0		0		0			0	X = 47.68	
															
	Yes	23 (15.33%)	1 (	0.98%)	3 (	3.95%)		0	8 (14.81%)		0	35 (	6.07%)		
											
	No	127 (84.67%)	101 (99.02%)	73 (96.05%)	111 (100.00%)	46 (85.19%)	84 (100.00%)	542	(93.93%)		
																		
Rash	Missing		0		0		0		0		0		0			0	X = 47.12	
																
	Yes	21 (14.00%)		0	1 (	1.32%)	1 (	0.90%)	2 (	3.70%)		0	25 (	4.33%)		
											
	No	129 (86.00%)	102 (100.00%)	75 (98.68%)	110 (99.10%)	52 (96.30%)	84 (100.00%)	552	(95.67%)		
																		
Buccal	Missing		0		0		0		0		0		0			0	X = 38.51	
mucositis																		
															
	Yes	19 (12.67%)		0	3 (	3.95%)	2 (	1.80%)	12 (22.22%)	6 (	7.14%)	42 (	7.28%)		
											
	No	131 (87.33%)	102 (100.00%)	73 (96.05%)	109 (98.20%)	42 (77.78%)	78 (92.86%)	535	(92.72%)		
																		
Nausea	Missing		0		0		0		0		0		0			0	X = 27.09	
											
	Yes	25 (16.67%)	16 (15.69%)	17 (22.37%)		0	12 (22.22%)	17 (20.24%)	87 (15.08%)		
											
	No	125 (83.33%)	86 (84.31%)	59 (77.63%)	111 (100.00%)	42 (77.78%)	67 (79.76%)	490	(84.92%)		
																		
Neutropenia	Missing		0		0		0		0		0		0			0	X = 10.74	
																		
														
	Yes	18 (12.00%)	14 (13.73%)	4 (	5.26%)	4 (	3.60%)	3 (	5.56%)	9 (10.71%)	52 (	9.01%)		
											
	No	132 (88.00%)	88 (86.27%)	72 (94.74%)	107 (96.40%)	51 (94.44%)	75 (89.29%)	525 	(90.99%)		
																		
Weight	Missing		0		0		0		0		0		0			0	X = 5.83	
loss																		
																	
	Yes	4 (	2.67%)	2 (	1.96%)	2 (	2.63%)		0	2 (	3.70%)		0	10 (	1.73%)		
											
	No	146 (97.33%)	100 (98.04%)	74 (97.37%)	111 (100.00%)	52 (96.30%)	84 (100.00%)	567	(98.27%)		
																		
Gastro	Missing		0		0		0		0		0		0			0	X = 10.55	
mucositis																		
															
	Yes	20 (13.33%)	12 (11.76%)	6 (	7.89%)	3 (	2.70%)	4 (	7.41%)	6 (	7.14%)	51 (	8.84%)		
											
	No	130 (86.67%)	90 (88.24%)	70 (92.11%)	108 (97.30%)	50 (92.59%)	78 (92.86%)	526	(91.16%)		
																		
Headache	Missing		0		0		0		0		0		0			0	X = 12.77	
																		
																	
	Yes	7 (	4.67%)	2 (	1.96%)	5 (	6.58%)		0	1 (	1.85%)		0	15 (	2.60%)		
											
	No	143 (95.33%)	100 (98.04%)	71 (93.42%)	111 (100.00%)	53 (98.15%)	84 (100.00%)	562	(97.40%)		
																		
Other	Missing		0		0		0		0		0		0			0	X = 43.21	
															
	Yes	17 (11.33%)	5 (	4.90%)	16 (21.05%)	1 (	0.90%)		0	1 (	1.19%)	40 (	6.93%)		
											
	No	133 (88.67%)	97 (95.10%)	60 (78.95%)	110 (99.10%)	54 (100.00%)	83 (98.81%)	537	(93.07%)		
																		


Table B6. Home care

					Drôme				
		Pôle Lille	Paul D'Egine	Clairval	Ardèche	Peupliers	Jean Mermoz	total	
									
Home-care	Missing	0	0	0	0	0	0	0	
									
	Valid	150	102	76	111	54	84	577	
	values								
									
	Yes	95 (63.33%)	64 (62.75%)	61 (80.26%)	0	32 (59.26%)	0	252 (43.67%)	
									
	No	55 (36.67%)	38 (37.25%)	15 (19.74%)	111 (100.00%)	22 (40.74%)	84 (100.00%)	325 (56.33%)	
									


Table B7. Hospitalization characteristics

					Drôme					
		Pôle Lille	Paul D'Egine	Clairval	Ardèche	Peupliers	Jean Mermoz		total	
											
Hospitalization	Missing	0	1 (9.09%)	0		0	0	0	1 (	3.13%)	
service											
											
	Valid	4	10	2		12	1	2		31	
	values										
											
	Cardiologie	1 (25.00%)	0	1 (50.00%)		0	0	0	2 (	6.45%)	
									
	Chirurgie	3 (75.00%)	0	0	4 (33.33%)	1 (100.00%)	0	8 (25.81%)	
									
	Medecine	0	1 (10.00%)	0	6 (50.00%)	0	0	7 (22.58%)	
										
	Oncologie	0	6 (60.00%)	1 (50.00%)		0	0	0	7 (22.58%)	
											
	Soins	0	0	0	1 (	8.33%)	0	0	1 (	3.23%)	
	continus										
										
	Urgence	0	3 (30.00%)	0	1 (	8.33%)	0	2 (100.00%)	6 (19.35%)	
											

Table B8. Income for hospital per cycle

			Paul		Drôme		Jean			
		Pôle Lille	D'Egine	Clairval	Ardèche	Peupliers	Mermoz	total	p-value	
										
Administration	Missing	0	0	0	0	0	0	0	W = 26.97	
chemotherapy										
										
	Nb of valid	150	102	76	111	54	84	577	p < 0.0001	
	values									
										
	Mean (Sd)	274.21	272.48	272.48	321.58	277.29	285.46	284.71		
		(21.20)	(0.00)	(0.00)	(156.74)	(35.34)	(83.58)	(79.19)		
										
	Min-Max	[272.5;	[272.5;	[272.5;	[272.5;	[272.5;	[272.5;	[272.5;		
		532.2]	272.5]	272.5]	817.4]	532.2]	817.4]	817.4]		
										
	Median	272.5	272.5	272.5	272.5	272.5	272.5	272.5		
										
Chemotherapy	Missing	0	0	0	0	0	0	0	W = 106.69	
drug										
										
	Nb of valid	150	102	76	111	54	84	577	p < 0.0001	
	values									
										
	Mean (Sd)	50.45	75.00	54.95	50.23	52.63	50.89	55.61		
		(25.00)	(0.00)	(19.47)	(21.71)	(23.67)	(16.21)	(21.77)		
										
	Min-Max	[25.0;75.0]	[75.0;75.0]	[25.0;92.0]	[25.0;75.0]	[25.0;75.0]	[25.0;75.0]	[25.0;92.0]		
										
	Median	75.0	75.0	50.0	50.0	62.5	50.0	75.0		
										
Symptomatic	Missing	0	0	0	0	0	0	0	T = 18.849	
prescription										
										
	Nb of valid	150	102	76	111	54	84	577	p < 0.0001	
	values									
										
	Mean (Sd)	21.18	85.95	32.78	30.61	71.78	21.18	40.71		
		(9.17)	(43.30)	(14.59)	(133.16)	(30.63)	(1.95)	(66.99)		
										
	Min-Max	[6.8;40.9]	[31.7;	[19.7;	[0.0;	[31.7;	[11.0;24.2]	[0.0;		
			157.6]	102.3]	1007.1]	121.2]		1007.1]		
										
	Median	21.0	101.6	31.7	21.0	63.4	21.0	24.2		
										
Side effect	Missing	0	0	0	0	0	0	0	W = 18.70	
management										
										
	Nb of valid	150	102	76	111	54	84	577	p = 0.002	
	values									
										
	Mean (Sd)	10.08	281.88	63.06	251.42	2.32	2.35	109.68		
		(94.21)	(1104.68)	(549.74)	(1126.68)	(17.06)	(18.40)	(716.15)		
										
	Min-Max	[0.0;	[0.0;	[0.0;	[0.0;	[0.0;125.4]	[0.0;166.0]	[0.0;		
		1136.0]	5970.2]	4792.5]	7145.0]			7145.0]		
										
	Median	0.0	0.0	0.0	0.0	0.0	0.0	0.0		
										
Monitoring	Missing	0	0	0	0	2 (3.7%)	0	2 (0.3%)	W = 81.59	
										
	Nb of valid	150	102	76	111	52	84	575	p < 0.0001	
	values									
										
	Mean (Sd)	20.47	25.48	25.42	38.25	25.65	24.00	26.43		
		(13.86)	(14.61)	(8.15)	(19.15)	(11.75)	(11.62)	(15.35)		
										
	Min-Max	[0.0;56.0]	[0.0;79.0]	[0.0;28.0]	[0.0;112.0]	[0.0;56.0]	[0.0;56.0]	[0.0;112.0]		
										
	Median	28.0	28.0	28.0	28.0	28.0	28.0	28.0		
										
Transport	Missing	0	0	0	0	2 (3.7%)	0	2 (0.3%)	W = 0.00	
										
	Nb of valid	150	102	76	111	52	84	575	p = 1.00	
	values									
										
	Mean (Sd)	0.00 (0.00)	0.00 (0.00)	0.00 (0.00)	0.00 (0.00)	0.00 (0.00)	0.00 (0.00)	0.00 (0.00)		
										
	Min-Max	[0.0;0.0]	[0.0;0.0]	[0.0;0.0]	[0.0;0.0]	[0.0;0.0]	[0.0;0.0]	[0.0;0.0]		
										
	Median	0.0	0.0	0.0	0.0	0.0	0.0	0.0		
										
Absenteism	Missing	0	0	0	0	0	0	0	W = 0.00	
										
	Nb of valid	150	102	76	111	54	84	577	p = 1.00	
	values									
										
	Mean (Sd)	0.00 (0.00)	0.00 (0.00)	0.00 (0.00)	0.00 (0.00)	0.00 (0.00)	0.00 (0.00)	0.00 (0.00)		
										
	Min-Max	[0.0;0.0]	[0.0;0.0]	[0.0;0.0]	[0.0;0.0]	[0.0;0.0]	[0.0;0.0]	[0.0;0.0]		
										
	Median	0.0	0.0	0.0	0.0	0.0	0.0	0.0		
										
Total	Missing	0	0	0	0	2 (3.7%)	0	2 (0.3%)	W = 175.15	
										
	Nb of valid	150	102	76	111	52	84	575	p < 0.0001	
	values									
										
	Mean (Sd)	376.39	740.78	448.69	692.09	429.79	383.88	517.45		
		(100.44)	(1109.42)	(552.98)	(1190.41)	(60.37)	(91.33)	(745.74)		
										
	Min-Max	[304.2;	[379.2;	[323.0;	[297.5;	[332.4;	[308.5;	[297.5;		
		1468.2]	6502.8]	5199.7]	7610.6]	648.0]	942.1]	7610.6]		
										
	Median	370.2	477.1	382.2	396.5	431.3	371.5	396.5		
										

Table B9. Income for hospital by patient


			Paul		Drôme		Jean			
		Pôle Lille	D'Egine	Clairval	Ardèche	Peupliers	Mermoz	total	p-value	
										
Administration	Missing	0	0	0	0	0	0	0	W = 26.35	
chemotherapy										
										
	Nb of valid	25	17	16	21	9	18	106	p < 0.0001	
	values									
										
	Mean (Sd)	1645.27	1634.88	1294.28	1699.76	1663.74	1332.12	1549.81		
		(51.94)	(0.00)	(322.40)	(583.80)	(285.90)	(466.26)	(385.79)		
										
	Min-Max	[1634.9;	[1634.9;	[817.4;	[1089.9;	[1089.9;	[545.0;	[545.0;		
		1894.6]	1634.9]	1634.9]	2724.8]	2179.8]	2724.8]	2724.8]		
										
	Median	1634.9	1634.9	1089.9	1634.9	1634.9	1089.9	1634.9		
										
Chemotherapy	Missing	0	0	0	0	0	0	0	W = 60.75	
drug										
										
	Nb of valid	25	17	16	21	9	18	106	p < 0.0001	
	values									
										
	Mean (Sd)	302.68	450.00	261.00	265.48	315.78	237.50	302.69		
		(22.92)	(0.00)	(76.98)	(47.75)	(64.88)	(68.73)	(85.35)		
										
	Min-Max	[250.0;	[450.0;	[150.0;	[200.0;	[200.0;	[100.0;	[100.0;		
		400.0]	450.0]	450.0]	300.0]	425.0]	400.0]	450.0]		
										
	Median	300.0	450.0	262.5	300.0	300.0	200.0	300.0		
										
Symptomatic	Missing	0	0	0	0	0	0	0	T = 15.475	
prescription										
										
	Nb of valid	25	17	16	21	9	18	106	p < 0.0001	
	values									
										
	Mean (Sd)	127.10	515.68	155.70	161.80	430.67	98.86	221.59		
		(39.47)	(244.02)	(69.29)	(314.35)	(151.98)	(25.77)	(233.75)		
										
	Min-Max	[83.2;	[192.4;	[69.5;	[0.0;	[183.2;	[42.0;	[0.0;		
		180.0]	868.1]	383.4]	1129.8]	691.4]	142.1]	1129.8]		
										
	Median	136.0	606.2	127.8	62.9	438.4	83.9	129.0		
										
Side effect	Missing	0	0	0	0	0	0	0	T = 2.233	
management										
										
	Nb of valid	25	17	16	21	9	18	106	p = 0.06	
	values									
										
	Mean (Sd)	60.49	1691.27	299.53	1328.95	13.93	10.98	597.05		
		(227.87)	(3877.68)	(1198.13)	(2996.72)	(41.80)	(39.39)	(2165.68)		
										
	Min-Max	[0.0;	[0.0;	[0.0;	[0.0;	[0.0;125.4]	[0.0;166.0]	[0.0;		
		1136.0]	14799.4]	4792.5]	11604.9]			14799.4]		
										
	Median	0.0	0.0	0.0	0.0	0.0	0.0	0.0		
										
Monitoring	Missing	0	0	0	0	0	0	0	W = 35.71	
										
	Nb of valid	25	17	16	21	9	18	106	p < 0.0001	
	values									
										
	Mean (Sd)	122.80	152.88	120.75	202.19	154.44	112.00	143.90		
		(23.75)	(59.66)	(33.47)	(60.48)	(41.53)	(35.93)	(54.16)		
										
	Min-Max	[84.0;	[28.0;	[56.0;	[112.0;	[107.0;	[28.0;	[28.0;		
		196.0]	275.0]	168.0]	308.0]	247.0]	168.0]	308.0]		
										
	Median	112.0	168.0	112.0	196.0	140.0	112.0	140.0		
										
Transport	Missing	0	0	0	0	0	0	0	W = 0.00	
										
	Nb of valid	25	17	16	21	9	18	106	p = 1.00	
	values									
										
	Mean (Sd)	0.00 (0.00)	0.00 (0.00)	0.00 (0.00)	0.00 (0.00)	0.00 (0.00)	0.00 (0.00)	0.00 (0.00)		
										
	Min-Max	[0.0;0.0]	[0.0;0.0]	[0.0;0.0]	[0.0;0.0]	[0.0;0.0]	[0.0;0.0]	[0.0;0.0]		
										
	Median	0.0	0.0	0.0	0.0	0.0	0.0	0.0		
										
Absenteeism	Missing	0	0	0	0	0	0	0	W = 0.00	
										
	Nb of valid	25	17	16	21	9	18	106	p = 1.00	
	values									
										
	Mean (Sd)	0.00 (0.00)	0.00 (0.00)	0.00 (0.00)	0.00 (0.00)	0.00 (0.00)	0.00 (0.00)	0.00 (0.00)		
										
	Min-Max	[0.0;0.0]	[0.0;0.0]	[0.0;0.0]	[0.0;0.0]	[0.0;0.0]	[0.0;0.0]	[0.0;0.0]		
										
	Median	0.0	0.0	0.0	0.0	0.0	0.0	0.0		
										
Total	Missing	0	0	0	0	0	0	0	T = 3.969	
										
	Nb of valid	25	17	16	21	9	18	106	p = 0.003	
	values									
										
	Mean (Sd)	2258.34	4444.71	2131.26	3658.17	2578.57	1791.46	2815.03		
		(227.24)	(3852.94)	(1244.66)	(3217.14)	(496.60)	(596.70)	(2333.39)		
										
	Min-Max	[2102.1;	[2305.3;	[1092.9;	[1429.9;	[1585.1;	[714.9;	[714.9;		
		3270.7]	17302.8]	6472.3]	14615.8]	3518.3]	3420.0]	17302.8]		
										
	Median	2210.9	2947.1	1888.7	2500.6	2536.8	1485.8	2246.5		
										


Table B10. Aggregated costs per patient - Insurance perspective

			Paul		Drôme		Jean			
		Pôle Lille	D'Egine	Clairval	Ardèche	Peupliers	Mermoz	total	p-value	
										
Administration	Missing	0	0	0	0	0	0	0	W = 26.35	
chemotherapy										
										
	Nb of valid	25	17	16	21	9	18	106	p < 0.0001	
	values									
										
	Mean (Sd)	1645.27	1634.88	1294.28	1699.76	1663.74	1332.12	1549.81		
		(51.94)	(0.00)	(322.40)	(583.80)	(285.90)	(466.26)	(385.79)		
										
	Min-Max	[1634.9;	[1634.9;	[817.4;	[1089.9;	[1089.9;	[545.0;	[545.0;		
		1894.6]	1634.9]	1634.9]	2724.8]	2179.8]	2724.8]	2724.8]		
										
	Median	1634.9	1634.9	1089.9	1634.9	1634.9	1089.9	1634.9		
										
Chemotherapy	Missing	0	0	0	0	0	0	0	W = 60.75	
drug										
										
	Nb of valid	25	17	16	21	9	18	106	p < 0.0001	
	values									
										
	Mean (Sd)	302.68	450.00	261.00	265.48	315.78	237.50	302.69		
		(22.92)	(0.00)	(76.98)	(47.75)	(64.88)	(68.73)	(85.35)		
										
	Min-Max	[250.0;	[450.0;	[150.0;	[200.0;	[200.0;	[100.0;	[100.0;		
		400.0]	450.0]	450.0]	300.0]	425.0]	400.0]	450.0]		
										
	Median	300.0	450.0	262.5	300.0	300.0	200.0	300.0		
										
Symptomatic	Missing	0	0	0	0	0	0	0	W = 49.84	
prescription										
										
	Nb of valid	25	17	16	21	9	18	106	p < 0.0001	
	values									
										
	Mean (Sd)	3863.16	1815.43	3565.35	161.80	2797.06	2534.64	2440.39		
		(2376.07)	(1005.60)	(2171.31)	(314.35)	(2132.96)	(2208.90)	(2249.97)		
										
	Min-Max	[298.8;	[697.8;	[473.7;	[0.0;	[183.2;	[614.3;	[0.0;		
		6388.9]	4281.0]	6571.3]	1129.8]	5795.1]	7384.9]	7384.9]		
										
	Median	5272.3	1383.0	3257.9	62.9	2931.5	1506.4	1580.7		
										
Side effect	Missing	0	0	0	0	0	0	0	T = 2.663	
management										
										
	Nb of valid	25	17	16	21	9	18	106	p = 0.03	
	values									
										
	Mean (Sd)	94.94	2023.61	349.38	1333.38	33.69	123.36	687.64		
		(231.28)	(3881.11)	(1221.04)	(2995.69)	(73.90)	(263.11)	(2192.31)		
										
	Min-Max	[0.0;	[0.0;	[0.0;	[0.0;	[0.0;228.9]	[0.0;	[0.0;		
		1164.0]	14989.5]	4922.1]	11604.9]		1013.2]	14989.5]		
										
	Median	31.7	55.6	17.9	3.7	7.4	0.0	15.9		
										
Monitoring	Missing	0	0	0	0	0	0	0	T = 26.682	
										
	Nb of valid	25	17	16	21	9	18	106	p < 0.0001	
	values									
										
	Mean (Sd)	640.86	833.49	522.96	506.28	677.84	559.54	616.62		
		(57.54)	(133.81)	(76.87)	(86.92)	(96.18)	(132.11)	(147.33)		
										
	Min-Max	[553.6;	[432.7;	[376.6;	[390.5;	[534.9;	[351.6;	[351.6;		
		827.0]	1020.5]	648.9]	702.3]	813.6]	903.0]	1020.5]		
										
	Median	635.2	838.7	524.1	481.1	678.1	531.8	603.8		
										
Transport	Missing	0	0	0	0	0	0	0	W = 5.83	
										
	Nb of valid	25	17	16	21	9	18	106	p = 0.32	
	values									
										
	Mean (Sd)	469.94	618.09	739.90	730.94	885.88	489.78	624.84		
		(250.67)	(343.61)	(528.87)	(544.05)	(1042.41)	(444.82)	(512.32)		
										
	Min-Max	[167.9;	[192.7;	[101.7;	[122.8;	[122.8;	[122.8;	[101.7;		
		1298.7]	1296.9]	1884.4]	2295.1]	3472.0]	1710.7]	3472.0]		
										
	Median	417.4	588.2	722.8	608.4	533.1	282.3	437.4		
										
Absenteeism	Missing	0	0	0	0	0	0	0	T = 1.645	
										
	Nb of valid	25	17	16	21	9	18	106	p = 0.16	
	values									
										
	Mean (Sd)	1650.31	2471.16	2068.66	1515.50	3147.19	1949.94	1996.37		
		(1606.93)	(1881.24)	(1532.92)	(1832.89)	(1513.34)	(1700.06)	(1724.38)		
										
	Min-Max	[0.0;	[0.0;	[0.0;	[0.0;	[0.0;	[0.0;	[0.0;		
		3819.1]	3819.1]	3819.1]	4243.4]	5092.1]	4182.8]	5092.1]		
										
	Median	1996.4	3819.1	2546.0	0.0	3819.1	2546.0	2546.0		
										
Total	Missing	0	0	0	0	0	0	0	T = 2.599	
										
	Nb of valid	25	17	16	21	9	18	106	p = 0.03	
	values									
										
	Mean (Sd)	8667.16	9846.65	8801.52	6213.13	9521.18	7226.89	8218.37		
		(3039.84)	(4862.09)	(3132.61)	(3764.59)	(4184.05)	(3068.93)	(3784.40)		
										
	Min-Max	[3187.9;	[4701.9;	[4445.9;	[2228.9;	[4072.0;	[2535.5;	[2228.9;		
		13547.7]	23937.5]	14599.3]	17310.2]	15619.6]	12735.5]	23937.5]		
										
	Median	8405.1	8851.9	8212.8	4724.4	10339.1	7478.1	8193.2		
										
Total without	Missing	0	0	0	0	0	0	0	T = 2.255	
absenteeism										
										
	Nb of valid	25	17	16	21	9	18	106	p = 0.055	
	values									
										
	Mean (Sd)	7016.85	7375.50	6732.87	4697.63	6373.99	5276.94	6222.00		
		(2379.05)	(4099.90)	(2691.45)	(3628.21)	(3221.91)	(2365.05)	(3181.09)		
										
	Min-Max	[3187.9;	[4326.7;	[3181.2;	[1803.3;	[2162.5;	[2491.6;	[1803.3;		
		9728.7]	20118.4]	12053.3]	17310.2]	11800.5]	10113.8]	20118.4]		
										
	Median	8262.5	5598.6	6003.7	3495.1	6520.0	4762.6	5583.5		
										


Table B11. Aggregated costs per cycle - Insurance perspective

			Paul		Drôme		Jean			
		Pôle Lille	D'Egine	Clairval	Ardèche	Peupliers	Mermoz	total	p-value	
										
Administration	Missing	0	0	0	0	0	0	0	W = 26.97	
chemotherapy										
										
	Nb of valid	150	102	76	111	54	84	577	p < 0.0001	
	values									
										
	Mean (Sd)	274.21	272.48	272.48	321.58	277.29	285.46	284.71		
		(21.20)	(0.00)	(0.00)	(156.74)	(35.34)	(83.58)	(79.19)		
										
	Min-Max	[272.5;	[272.5;	[272.5;	[272.5;	[272.5;	[272.5;	[272.5;		
		532.2]	272.5]	272.5]	817.4]	532.2]	817.4]	817.4]		
										
	Median	272.5	272.5	272.5	272.5	272.5	272.5	272.5		
										
Chemotherapy	Missing	0	0	0	0	0	0	0	W = 106.69	
drug										
										
	Nb of valid	150	102	76	111	54	84	577	p < 0.0001	
	values									
										
	Mean (Sd)	50.45	75.00	54.95	50.23	52.63	50.89	55.61		
		(25.00)	(0.00)	(19.47)	(21.71)	(23.67)	(16.21)	(21.77)		
										
	Min-Max	[25.0;75.0]	[75.0;75.0]	[25.0;92.0]	[25.0;75.0]	[25.0;75.0]	[25.0;75.0]	[25.0;92.0]		
										
	Median	75.0	75.0	50.0	50.0	62.5	50.0	75.0		
										
Symptomatic	Missing	0	0	0	0	0	0	0	W = 277.57	
prescription										
										
	Nb of valid	150	102	76	111	54	84	577	p < 0.0001	
	values									
										
	Mean (Sd)	643.86	302.57	750.60	30.61	466.18	543.14	448.32		
		(478.67)	(256.00)	(452.59)	(133.16)	(372.01)	(533.79)	(464.06)		
										
	Min-Max	[6.8;	[31.7;	[31.7;	[0.0;	[45.8;	[50.6;	[0.0;		
		1093.3]	1039.5]	1839.5]	1007.1]	1086.4]	3104.6]	3104.6]		
										
	Median	996.1	217.2	1017.6	21.0	572.5	156.8	156.8		
										
Side effect	Missing	0	0	0	0	0	0	0	W = 8.29	
management										
										
	Nb of valid	150	102	76	111	54	84	577	p = 0.14	
	values									
										
	Mean (Sd)	15.82	337.27	73.55	252.26	5.62	26.43	126.32		
		(97.87)	(1119.77)	(558.23)	(1126.53)	(22.24)	(111.88)	(724.17)		
										
	Min-Max	[0.0;	[0.0;	[0.0;	[0.0;	[0.0;125.4]	[0.0;986.1]	[0.0;		
		1164.0]	6053.8]	4871.5]	7145.0]			7145.0]		
										
	Median	0.0	0.0	0.0	0.0	0.0	0.0	0.0		
										
Monitoring	Missing	0	0	0	0	2 (3.7%)	0	2 (0.3%)	T = 1.814	
										
	Nb of valid	150	102	76	111	52	84	575	p = 0.11	
	values									
										
	Mean (Sd)	106.81	138.91	110.10	95.78	115.60	119.90	113.52		
		(113.01)	(111.25)	(115.83)	(91.41)	(117.42)	(122.36)	(111.50)		
										
	Min-Max	[0.0;451.4]	[0.0;500.6]	[23.0;	[28.0;	[0.0;393.4]	[28.0;	[0.0;500.6]		
				392.3]	374.5]		421.7]			
										
	Median	64.2	108.6	55.8	45.3	64.2	64.2	64.2		
										
Transport	Missing	1 (0.7%)	0	0	0	2 (3.7%)	0	3 (0.5%)	W = 30.84	
										
	Nb of valid	149	102	76	111	52	84	574	p < 0.0001	
	values									
										
	Mean (Sd)	78.85	103.01	155.77	138.29	147.36	104.95	114.85		
		(47.13)	(59.57)	(120.79)	(123.55)	(165.58)	(107.54)	(104.67)		
										
	Min-Max	[22.6;	[32.1;	[33.9;	[24.0;	[30.7;	[24.0;	[22.6;		
		299.7]	299.4]	462.8]	580.8]	578.7]	427.7]	580.8]		
										
	Median	69.2	85.6	122.8	92.5	88.9	55.6	79.5		
										
Absenteism	Missing	36 (24.0%)	0	0	0	0	0	36 (6.2%)	W = 40.42	
										
	Nb of valid	114	102	76	111	54	84	541	p < 0.0001	
	values									
										
	Mean (Sd)	256.84	411.86	435.51	286.72	524.53	417.84	369.01		
		(302.00)	(305.68)	(297.83)	(333.20)	(224.25)	(315.66)	(315.53)		
										
	Min-Max	[0.0;636.5]	[0.0;636.5]	[0.0;636.5]	[0.0;848.7]	[0.0;636.5]	[0.0;818.4]	[0.0;848.7]		
										
	Median	0.0	636.5	636.5	0.0	636.5	636.5	636.5		
										
Total	Missing	37 (24.7%)	0	0	0	2 (3.7%)	0	39 (6.8%)	T = 5.29	
										
	Nb of valid	113	102	76	111	52	84	538	p < 0.0001	
	values									
										
	Mean (Sd)	1512.48	1641.11	1852.95	1175.46	1577.28	1548.62	1527.33		
		(578.15)	(1245.51)	(800.59)	(1273.28)	(581.57)	(612.58)	(957.41)		
										
	Min-Max	[356.2;	[505.9;	[1018.2;	[349.5;	[583.4;	[497.5;	[349.5;		
		3232.7]	7859.3]	7714.3]	8233.0]	2767.2]	3540.2]	8233.0]		
										
	Median	1520.6	1350.1	1703.3	1017.7	1606.8	1460.2	1425.2		
										


Table B12. Monitoring – Distribution of cycle with laboratory tests

								Drôme						
		Pôle Lille	Paul D'Egine	Clairval	Ardèche	Peupliers	Jean Mermoz		total	
											
Blood count	Missing	23 (15.33%)	5 (	4.90%)	11 (14.47%)	20 (18.02%)	8 (14.81%)	6 (	7.14%)	73 (12.65%)	
														
	Valid		127		97		65	91	46		78		504	
	values													
									
	Yes	127 (100.00%)	95 (97.94%)	65 (100.00%)	91 (100.00%)	46 (100.00%)	78 (100.00%)	502 (99.60%)	
														
	No		0	2 (	2.06%)		0	0	0		0	2 (	0.40%)	
											
Blood platelets	Missing	23 (15.33%)	5 (	4.90%)	11 (14.47%)	20 (18.02%)	8 (14.81%)	6 (	7.14%)	73 (12.65%)	
														
	Valid		127		97		65	91	46		78		504	
	values													
									
	Yes	127 (100.00%)	95 (97.94%)	65 (100.00%)	91 (100.00%)	46 (100.00%)	78 (100.00%)	502 (99.60%)	
														
	No		0	2 (	2.06%)		0	0	0		0	2 (	0.40%)	
										
Ionogramme	Missing	23 (15.33%)	5 (	4.90%)	11 (14.47%)	75 (67.57%)	8 (14.81%)	35 (41.67%)	157 (27.21%)	
														
	Valid		127		97		65	36	46		49		420	
	values													
									
	Yes	123 (96.85%)	11 (11.34%)	64 (98.46%)	2 (5.56%)	45 (97.83%)	40 (81.63%)	285 (67.86%)	
											
	No	4 (	3.15%)	86 (88.66%)	1 (	1.54%)	34 (94.44%)	1 (2.17%)	9 (18.37%)	135 (32.14%)	
										
Urea, creatine	Missing	23 (15.33%)	5 (	4.90%)	11 (14.47%)	75 (67.57%)	9 (16.67%)	35 (41.67%)	158 (27.38%)	
	Valid		127		97		65	36	45		49		419	
	values													
									
	Yes	124 (97.64%)	35 (36.08%)	64 (98.46%)	29 (80.56%)	34 (75.56%)	40 (81.63%)	326 (77.80%)	
											
	No	3 (	2.36%)	62 (63.92%)	1 (	1.54%)	7 (19.44%)	11 (24.44%)	9 (18.37%)	93 (22.20%)	
										
Hepatic test	Missing	23 (15.33%)	5 (	4.90%)	18 (23.68%)	72 (64.86%)	9 (16.67%)	35 (41.67%)	162 (28.08%)	
														
	Valid		127		97		58	39	45		49		415	
	values													
														
	Yes	121 (95.28%)	35 (36.08%)	7 (12.07%)	28 (71.79%)	43 (95.56%)	40 (81.63%)	274 (66.02%)	
										
	No	6 (	4.72%)	62 (63.92%)	51 (87.93%)	11 (28.21%)	2 (4.44%)	9 (18.37%)	141 (33.98%)	
										
Cytotoxic test	Missing	22 (14.67%)	6 (	5.88%)	19 (25.00%)	72 (64.86%)	8 (14.81%)	33 (39.29%)	160 (27.73%)	
													
	Valid		128		96	57		39	46	51		417	
	values												
													
	Yes		0	1 (	1.04%)	0	1 (	2.56%)	0	0	2 (	0.48%)	
									
	No	128 (100.00%)	95 (98.96%)	57 (100.00%)	38 (97.44%)	46 (100.00%)	51 (100.00%)	415 (99.52%)	
										
CRP test	Missing	22 (14.67%)	5 (	4.90%)	19 (25.00%)	72 (64.86%)	8 (14.81%)	35 (41.67%)	161 (27.90%)	
													
	Valid		128		97	57		39	46	49		416	
	values												
											
	Yes	3 (	2.34%)	37 (38.14%)	0	2 (	5.13%)	0	3 (6.12%)	45 (10.82%)	
									
	No	125 (97.66%)	60 (61.86%)	57 (100.00%)	37 (94.87%)	46 (100.00%)	46 (93.88%)	371 (89.18%)	
									
Other test	Missing	32 (21.33%)	41 (40.20%)	22 (28.95%)	75 (67.57%)	13 (24.07%)	33 (39.29%)	216 (37.44%)	
													
	Valid		118		61	54		36	41	51		361	
	values												
													
	Yes		0		0	0		0	0	9 (17.65%)	9 (	2.49%)	
									
	No	118 (100.00%)	61 (100.00%)	54 (100.00%)	36 (100.00%)	41 (100.00%)	42 (82.35%)	352 (97.51%)	
													


Table B13. Monitoring – Consultation per cycle

					Drôme				
		Pôle Lille	Paul D'Egine	Clairval	Ardèche	Peupliers	Jean Mermoz	total	
									
Oncologist	Missing	0	0	0	0	0	0	0	
									
	Mean (Sd)	0.91 (0.42)	0.95 (0.47)	1.00 (0.00)	1.16 (0.51)	0.94 (0.23)	1.01 (0.11)	0.99 (0.39)	
									
	Min-Max	[0.0;3.0]	[0.0;2.0]	[1.0;1.0]	[1.0;3.0]	[0.0;1.0]	[1.0;2.0]	[0.0;3.0]	
									
Generalist	Missing	0	0	0	0	0	0	0	
									
	Mean (Sd)	0.01 (0.12)	0.01 (0.10)	0.00 (0.00)	0.02 (0.13)	0.04 (0.19)	0.00 (0.00)	0.01 (0.11)	
									
	Min-Max	[0.0;1.0]	[0.0;1.0]	[0.0;0.0]	[0.0;1.0]	[0.0;1.0]	[0.0;0.0]	[0.0;1.0]	
									
Gynecologist	Missing	0	0	0	0	0	0	0	
									
	Mean (Sd)	0.00 (0.00)	0.00 (0.00)	0.00 (0.00)	0.21 (0.43)	0.02 (0.14)	0.04 (0.19)	0.05 (0.22)	
									
	Min-Max	[0.0;0.0]	[0.0;0.0]	[0.0;0.0]	[0.0;2.0]	[0.0;1.0]	[0.0;1.0]	[0.0;2.0]	
									
Cardiologist	Missing	0	0	0	0	0	0	0	
									
	Mean (Sd)	0.01 (0.12)	0.01 (0.10)	0.08 (0.27)	0.00 (0.00)	0.02 (0.14)	0.00 (0.00)	0.02 (0.13)	
									
	Min-Max	[0.0;1.0]	[0.0;1.0]	[0.0;1.0]	[0.0;0.0]	[0.0;1.0]	[0.0;0.0]	[0.0;1.0]	
									
Radiologist	Missing	0	0	0	0	0	0	0	
									
	Mean (Sd)	0.14 (0.35)	0.01 (0.10)	0.00 (0.00)	0.01 (0.09)	0.04 (0.19)	0.15 (0.36)	0.07 (0.25)	
									
	Min-Max	[0.0;1.0]	[0.0;1.0]	[0.0;0.0]	[0.0;1.0]	[0.0;1.0]	[0.0;1.0]	[0.0;1.0]	
									
Other	Missing	0	0	0	0	0	0	0	
specialist									
									
	Mean (Sd)	0.01 (0.08)	0.02 (0.14)	0.01 (0.11)	0.01 (0.09)	0.02 (0.14)	0.01 (0.11)	0.01 (0.11)	
									
	Min-Max	[0.0;1.0]	[0.0;1.0]	[0.0;1.0]	[0.0;1.0]	[0.0;1.0]	[0.0;1.0]	[0.0;1.0]	
									


Table B14. Pre-chemotherapy tests

			Paul		Drôme		Jean		
		Pôle Lille	D'Egine	Clairval	Ardèche	Peupliers	Mermoz	Total	
									
Implantable chamber	Missing	0	3 (17.65%)	0	0	1 (11.11%)	0	4 (3.77%)	
									
									
	Valid	25	14	16	21	8	18	102	
	values								
									
	Yes	25	14	16	21 (100.00%)	8	18 (100.00%)	102 (100.00%)	
		(100.00%)	(100.00%)	(100.00%)		(100.00%)			
									
Electrocardiogram	Missing	0	3 (17.65%)	7 (43.75%)	4 (19.05%)	0	3 (16.67%)	17 (16.04%)	
									
	Valid	25	14	9	17	9	15	89	
	values								
									
	Yes	3 (12.00%)	2 (14.29%)	3 (33.33%)	0	1 (11.11%)	15 (100.00%)	24 (26.97%)	
									
									
	No	22 (88.00%)	12 (85.71%)	6 (66.67%)	17 (100.00%)	8 (88.89%)	0	65 (73.03%)	
									
									
Thoracic radiography	Missing	0	9 (52.94%)	0	0	2 (22.22%)	0	11 (10.38%)	
									
									
	Valid	25	8	16	21	7	18	95	
	values								
									
	Yes	25	8 
(100.00%)	16	21 (100.00%)	7	18 (100.00%)	95 (100.00%)	
		(100.00%)		(100.00%)		(100.00%)			
									
Heart scan	Missing	0	3 (17.65%)	6 (37.50%)	4 (19.05%)	0	7 (38.89%)	20 (18.87%)	
									
	Valid	25	14	10	17	9	11	86	
	values								
									
	Yes	12 (	14	9 (90.00%)	1 (5.88%)	9	8 (72.73%)	53 (61.63%)	
		48.00%)	(100.00%)			(100.00%)			
									
	No	13 (	0	1 (10.00%)	16 (94.12%)	0	3 (27.27%)	33 (38.37%)	
		52.00%)							
									
Oncologist	Missing	0	0	0	0	0	0	0	
consultation									
									
	Valid	25	17	16	21	9	18	106	
	values								
									
	Yes	25	17	16	21 (100.00%)	9	18 (100.00%)	106 (100.00%)	
		(100.00%)	(100.00%)	(100.00%)		(100.00%)				
										
Laboratory test	Missing	0	0	0	0	0	1 (5.56%)	1 (	0.94%)	
										
	Valid	25	17	16	21	9	17		105	
	values									
									
	Yes	25	16 (94.12%)	16	17 (80.95%)	9	17 (100.00%)	100 (95.24%)	
		(100.00%)		(100.00%)		(100.00%)				
										
	No	0	1 (5.88%)	0	4 (19.05%)	0	0	5 (	4.76%)	
										


Table B15. Protocols – Number of cycles

					Drôme							
		Pôle Lille	Paul D'Egine	Clairval	Ardèche	Peupliers	Jean Mermoz	Total	
													
Protocol	Missing	0	0	0		0		0		0		0	
													
	Valid values	150	102	76		111		54		84		577	
									
	Doc	73 (48.67%)	0	15 (19.74%)	33 (29.73%)	22 (40.74%)	16 (19.05%)	159 (27.56%)	
										
	Doc+cyc	0	0	0	26 (23.42%)	4 (	7.41%)	48 (57.14%)	78 (13.52%)	
													
	Doc+epi+cyc	0	18 (17.65%)	0		0		0		0	18 (	3.12%)	
													
	Dox+cyc	0	0	15 (19.74%)		0		0		0	15 (	2.60%)	
													
	Epi	1 (0.67%)	0	0		0		0		0	1 (	0.17%)	
													
	Epi+cyc	0	0	18 (23.68%)		0	1 (	1.85%)		0	19 (	3.29%)	
									
	Epi+cyc+flu	76 (50.67%)	84 (82.35%)	28 (36.84%)	42 (37.84%)	27 (50.00%)	17 (20.24%)	274 (47.49%)	
													
	Epi+flu	0	0	0		0		0	1 (	1.19%)	1 (	0.17%)	
													
	Pac	0	0	0	8 (	7.21%)		0	2 (	2.38%)	10 (	1.73%)	
													
	Pac+cyc	0	0	0	2 (	1.80%)		0		0	2 (	0.35%)	
													

Table B16. Average cost by patient - Societal perspective

			Paul		Drôme		Jean			
		Pôle Lille	D'Egine	Clairval	Ardèche	Peupliers	Mermoz	total	p-value	
										
Administration	Missing	0	0	0	0	0	0	0	W = 26.35	
chemotherapy										
										
	Nb of valid	25	17	16	21	9	18	106	p < 0.0001	
	values									
										
	Mean (Sd)	1645.27	1634.88	1294.28	1699.76	1663.74	1332.12	1549.81		
		(51.94)	(0.00)	(322.40)	(583.80)	(285.90)	(466.26)	(385.79)		
										
	Min-Max	[1634.9;	[1634.9;	[817.4;	[1089.9;	[1089.9;	[545.0;	[545.0;		
		1894.6]	1634.9]	1634.9]	2724.8]	2179.8]	2724.8]	2724.8]		
										
	Median	1634.9	1634.9	1089.9	1634.9	1634.9	1089.9	1634.9		
										
Chemotherapy	Missing	0	0	0	0	0	0	0	W = 60.75	
drug										
										
	Nb of valid	25	17	16	21	9	18	106	p < 0.0001	
	values									
										
	Mean (Sd)	302.68	450.00	261.00	265.48	315.78	237.50	302.69		
		(22.92)	(0.00)	(76.98)	(47.75)	(64.88)	(68.73)	(85.35)		
										
	Min-Max	[250.0;	[450.0;	[150.0;	[200.0;	[200.0;	[100.0;	[100.0;		
		400.0]	450.0]	450.0]	300.0]	425.0]	400.0]	450.0]		
										
	Median	300.0	450.0	262.5	300.0	300.0	200.0	300.0		
										
Symptomatic	Missing	0	0	0	0	0	0	0	W = 49.84	
prescription										
										
	Nb of valid	25	17	16	21	9	18	106	p < 0.0001	
	values									
										
	Mean (Sd)	3863.16	1815.43	3565.35	161.80	2797.06	2534.64	2440.39		
		(2376.07)	(1005.60)	(2171.31)	(314.35)	(2132.96)	(2208.90)	(2249.97)		
										
	Min-Max	[298.8;	[697.8;	[473.7;	[0.0;	[183.2;	[614.3;	[0.0;		
		6388.9]	4281.0]	6571.3]	1129.8]	5795.1]	7384.9]	7384.9]		
										
	Median	5272.3	1383.0	3257.9	62.9	2931.5	1506.4	1580.7		
										
Side effect	Missing	0	0	0	0	0	0	0	T = 2.663	
management										
										
	Nb of valid	25	17	16	21	9	18	106	p = 0.03	
	values									
										
	Mean (Sd)	94.94	2023.61	349.38	1333.38	33.69	123.36	687.64		
		(231.28)	(3881.11)	(1221.04)	(2995.69)	(73.90)	(263.11)	(2192.31)		
										
	Min-Max	[0.0;	[0.0;	[0.0;	[0.0;	[0.0;228.9]	[0.0;	[0.0;		
		1164.0]	14989.5]	4922.1]	11604.9]		1013.2]	14989.5]		
										
	Median	31.7	55.6	17.9	3.7	7.4	0.0	15.9		
										
Monitoring	Missing	0	0	0	0	0	0	0	T = 26.682	
										
	Nb of valid	25	17	16	21	9	18	106	p < 0.0001	
	values									
										
	Mean (Sd)	640.86	833.49	522.96	506.28	677.84	559.54	616.62		
		(57.54)	(133.81)	(76.87)	(86.92)	(96.18)	(132.11)	(147.33)		
										
	Min-Max	[553.6;	[432.7;	[376.6;	[390.5;	[534.9;	[351.6;	[351.6;		
		827.0]	1020.5]	648.9]	702.3]	813.6]	903.0]	1020.5]		
										
	Median	635.2	838.7	524.1	481.1	678.1	531.8	603.8		
										
Transport	Missing	0	0	0	0	0	0	0	W = 4.12	
										
	Nb of valid	25	17	16	21	9	18	106	p = 0.53	
	values									
										
	Mean (Sd)	553.04	660.86	746.69	845.75	1112.13	608.64	714.46		
		(321.04)	(361.42)	(533.94)	(606.72)	(1342.10)	(571.87)	(608.48)		
										
	Min-Max	[179.2;	[221.5;	[102.0;	[139.9;	[139.9;	[139.9;	[102.0;		
		1604.7]	1405.6]	1901.7]	2419.6]	4447.8]	2185.8]	4447.8]		
										
	Median	480.0	604.9	728.8	626.6	655.1	353.9	510.7		
										
Absenteeism	Missing	0	0	0	0	0	0	0	T = 1.645	
										
	Nb of valid	25	17	16	21	9	18	106	p = 0.16	
	values									
										
	Mean (Sd)	3301.16	4943.13	4138.00	3031.50	6295.41	3900.53	3993.39		
		(3214.38)	(3763.10)	(3066.34)	(3666.39)	(3027.18)	(3400.69)	(3449.32)		
										
	Min-Max	[0.0;	[0.0;	[0.0;	[0.0;	[0.0;	[0.0;	[0.0;		
		7639.4]	7639.4]	7639.4]	8488.2]	10185.8]	8366.9]	10185.8]		
										
	Median	3993.4	7639.4	5092.9	0.0	7639.4	5092.9	5092.9		
										
Total	Missing	0	0	0	0	0	0	0	T = 2.442	
										
	Nb of valid	25	17	16	21	9	18	106	p = 0.04	
	values									
										
	Mean (Sd)	10401.10	12361.40	10877.65	7843.94	12895.65	9296.33	10305.01		
		(4227.65)	(6172.66)	(4132.63)	(4726.25)	(5587.47)	(4397.01)	(4979.21)		
										
	Min-Max	[3200.7;	[4730.8;	[4452.2;	[2231.1;	[5999.3;	[2565.5;	[2231.1;		
		17444.6]	28036.0]	17438.4]	17434.7]	20898.3]	16630.7]	28036.0]		
										
	Median	8531.4	12159.0	9446.3	7356.8	14204.3	9386.3	9448.0		
										
Total without	Missing	0	0	0	0	0	0	0	T = 2.082	
absenteeism										
										
	Nb of valid	25	17	16	21	9	18	106	p = 0.07	
	values									
										
	Mean (Sd)	7099.95	7418.27	6739.65	4812.44	6600.24	5395.80	6311.62		
		(2377.44)	(4145.93)	(2691.22)	(3671.33)	(3469.34)	(2401.66)	(3215.39)		
										
	Min-Max	[3200.7;	[4457.0;	[3189.1;	[1820.4;	[2179.6;	[2508.7;	[1820.4;		
		9805.2]	20396.6]	12062.9]	17434.7]	12776.4]	10123.4]	20396.6]		
										
	Median	8352.0	5615.3	6008.3	3732.8	6564.9	4804.9	5591.9		
										


Table B17. Average cost per cycle – Societal perspective

			Paul		Drôme		Jean			
		Pôle Lille	D'Egine	Clairval	Ardèche	Peupliers	Mermoz	total	p-value	
										
Administration	Missing	0	0	0	0	0	0	0	W = 26.97	
chemotherapy										
										
	Nb of valid	150	102	76	111	54	84	577	p < 0.0001	
	values									
										
	Mean (Sd)	274.21	272.48	272.48	321.58	277.29	285.46	284.71		
		(21.20)	(0.00)	(0.00)	(156.74)	(35.34)	(83.58)	(79.19)		
										
	Min-Max	[272.5;	[272.5;	[272.5;	[272.5;	[272.5;	[272.5;	[272.5;		
		532.2]	272.5]	272.5]	817.4]	532.2]	817.4]	817.4]		
										
	Median	272.5	272.5	272.5	272.5	272.5	272.5	272.5		
										
Chemotherapy	Missing	0	0	0	0	0	0	0	W = 106.69	
drug										
										
	Nb of valid	150	102	76	111	54	84	577	p < 0.0001	
	values									
										
	Mean (Sd)	50.45	75.00	54.95	50.23	52.63	50.89	55.61		
		(25.00)	(0.00)	(19.47)	(21.71)	(23.67)	(16.21)	(21.77)		
										
	Min-Max	[25.0;75.0]	[75.0;75.0]	[25.0;92.0]	[25.0;75.0]	[25.0;75.0]	[25.0;75.0]	[25.0;92.0]		
										
	Median	75.0	75.0	50.0	50.0	62.5	50.0	75.0		
										
Symptomatic	Missing	0	0	0	0	0	0	0	W = 277.57	
prescription										
										
	Nb of valid	150	102	76	111	54	84	577	p < 0.0001	
	values									
										
	Mean (Sd)	643.86	302.57	750.60	30.61	466.18	543.14	448.32		
		(478.67)	(256.00)	(452.59)	(133.16)	(372.01)	(533.79)	(464.06)		
										
	Min-Max	[6.8;	[31.7;	[31.7;	[0.0;	[45.8;	[50.6;	[0.0;		
		1093.3]	1039.5]	1839.5]	1007.1]	1086.4]	3104.6]	3104.6]		
										
	Median	996.1	217.2	1017.6	21.0	572.5	156.8	156.8		
										
Side effect	Missing	0	0	0	0	0	0	0	W = 8.29	
management										
										
	Nb of valid	150	102	76	111	54	84	577	p = 0.14	
	values									
										
	Mean (Sd)	15.82	337.27	73.55	252.26	5.62	26.43	126.32		
		(97.87)	(1119.77)	(558.23)	(1126.53)	(22.24)	(111.88)	(724.17)		
										
	Min-Max	[0.0;	[0.0;	[0.0;	[0.0;	[0.0;125.4]	[0.0;986.1]	[0.0;		
		1164.0]	6053.8]	4871.5]	7145.0]			7145.0]		
										
	Median	0.0	0.0	0.0	0.0	0.0	0.0	0.0		
										
Monitoring	Missing	0	0	0	0	2 (3.7%)	0	2 (0.3%)	T = 1.814	
										
	Nb of valid	150	102	76	111	52	84	575	p = 0.11	
	values									
										
	Mean (Sd)	106.81	138.91	110.10	95.78	115.60	119.90	113.52		
		(113.01)	(111.25)	(115.83)	(91.41)	(117.42)	(122.36)	(111.50)		
										
	Min-Max	[0.0;451.4]	[0.0;500.6]	[23.0;	[28.0;	[0.0;393.4]	[28.0;	[0.0;500.6]		
				392.3]	374.5]		421.7]			
										
	Median	64.2	108.6	55.8	45.3	64.2	64.2	64.2		
										
Transport	Missing	1 (0.7%)	0	0	0	2 (3.7%)	0	3 (0.5%)	W = 19.10	
										
	Nb of valid	149	102	76	111	52	84	574	p = 0.002	
	values									
										
	Mean (Sd)	92.79	110.14	157.20	160.01	184.99	130.42	131.26		
		(59.39)	(64.06)	(121.98)	(139.38)	(213.34)	(137.89)	(122.85)		
										
	Min-Max	[23.7;	[36.9;	[34.0;	[25.6;	[35.0;	[25.6;	[23.7;		
		370.3]	324.5]	467.2]	637.8]	741.3]	546.4]	741.3]		
										
	Median	81.0	99.6	123.8	113.9	109.2	63.9	90.2		
										
Absenteism	Missing	36 (24.0%)	0	0	0	0	0	36 (6.2%)	W = 40.42	
										
	Nb of valid	114	102	76	111	54	84	541	p < 0.0001	
	values									
										
	Mean (Sd)	513.76	823.85	871.16	573.53	1049.24	835.83	738.15		
		(604.11)	(611.46)	(595.77)	(666.52)	(448.58)	(631.42)	(631.16)		
										
	Min-Max	[0.0;	[0.0;	[0.0;	[0.0;	[0.0;	[0.0;	[0.0;		
		1273.2]	1273.2]	1273.2]	1697.6]	1273.2]	1637.0]	1697.6]		
										
	Median	0.0	1273.2	1273.2	0.0	1273.2	1273.2	1273.2		
										
Total	Missing	37 (24.7%)	0	0	0	2 (3.7%)	0	39 (6.8%)	T = 6.509	
										
	Nb of valid	113	102	76	111	52	84	538	p < 0.0001	
	values									
										
	Mean (Sd)	1785.83	2060.23	2290.03	1483.99	2135.32	1992.07	1912.78		
		(807.35)	(1393.07)	(932.88)	(1376.03)	(765.30)	(812.04)	(1113.26)		
										
	Min-Max	[357.3;	[510.7;	[1021.1;	[351.1;	[624.1;	[509.3;	[351.1;		
		3884.4]	8521.1]	8355.9]	8290.0]	3566.6]	3541.8]	8521.1]		
										
	Median	1601.1	1964.3	2264.9	1126.3	2266.8	1961.0	1862.5		
										

Table B18. Distribution of chemotherapy strategies and protocols

				Paul		Drôme			Jean					
		Pôle Lille	D'Egine	Clairval	Ardèche	Peupliers	Mermoz		Total	test	
														
Chemotherapy	Missing	0		0	0	0	0		0		0		X =	
strategies													215.88	
												
	Valid values	25	17	16	21	9		18		106	p <	
													0.0001	
													
	DOC+CYC	0		0	0	6 (28.57%)	1 (	12 (66.67%)		19	(		
							11.11%)			17.92%)		
													
	DOC+CYC/PAC+CYC	0		0	0	1 (4.76%)	0		0	1 (	0.94%)		
													
	DOC+EPI+CYC	0		3 (17.65%)	0	0	0		0	3 (	2.83%)		
													
	DOC/EPI+CYC+FLU	24 (96.00%)	0	5 (31.25%)	10 (47.62%)	7 (77.78%)	4 (22.22%)	50(47.17%)		
											
											
	DOC/EPI+CYC+FLU/EPI+FLU	0		0	0	0	0	1 (	5.56%)	1 (	0.94%)		
														
													
	DOC/EPI+CYC+FLU/PAC	0		0	0	4 (19.05%)	0	1 (	5.56%)	5 (	4.72%)		
												
	DOC/EPI/EPI+CYC+FLU	1 (4.00%)	0	0	0	0		0	1 (	0.94%)		
													
	DOX+CYC	0		0	4 (	0	0		0	4 (	3.77%)		
					25.00%)									
													
	EPI+CYC	0		0	4 (	0	0		0	4 (	3.77%)		
					25.00%)									
														
	EPI+CYC+FLU	0		14 (82.35%)	3 (18.75%)	0	0		0	17(16.04%)		
												
													
	EPI+CYC/EPI+CYC+FLU	0		0	0	0	1 (11.11%)		0	1 (	0.94%)		
														
Distribution of chemotherapy protocols (for patient with at least one cycle under the protocol)	
														
	Missing	0		0	0	0	0		0		0			
												
	Valid values	51	17	21	40	17		26		172		
													
	Doc	25(49.02%)	0	5 (23.81%)	14 (35.00%)	7 (41.18%)	6 (23.08%)	57(33.14%)		
											
										
	Doc+cyc	0		0	0	7 (17.50%)	1 (5.88%)	12 (46.15%)		20	(		
										11.63%)		
													
	Doc+epi+cyc	0		3 (17.65%)	0	0	0		0	3 (	1.74%)		
														
	Dox+cyc	0		0	4 (19.05%)	0	0		0	4	(	2.33%)		
														
													
	Epi	1 (1.96%)	0	0	0	0		0	1	(	0.58%)		
														
	Epi+cyc	0		0	4 (
19.05%)	0	1 (
5.88%)		0	5	(	2.91%)		
														
												
	Epi+cyc+flu	25 (49.02%)	14 (82.35%)	8 (38.10%)	14 (35.00%)	8 (47.06%)	6 (23.08%)		75(43.60%)		
												
													
	Epi+flu	0		0	0	0	0	1 (	3.85%)	1	(	0.58%)		
														
	Pac	0		0	0	4 (10.00%)	0	1 (	3.85%)	5	(	2.91%)		
														
	Pac+cyc	0		0	0	1 (2.50%)	0		0	1	(	0.58%)		
														
Average number of cycle per protocol	
DOC	Missing	0		-	0	0	0		0			0	W = 11.22	
										
	Mean (Sd)	2.92 (0.40)	-	3.00 (0.00)	2.36 (0.93)	3.14 (0.38)	2.67 (0.82)	2.79 (0.65)		
										
	Min-Max	[1.0;3.0]	-	[3.0;3.0]	[1.0;3.0]	[3.0;4.0]	[1.0;3.0]	[1.0;4.0]		
														
DOC+CYC	Missing	-		-	-	0	0		0			0	T = 0.276	
											
	Mean (Sd)	-		-	-	3.71 (0.76)	4.00 (--)	4.00 (0.85)	3.90 (0.79)		
											
	Min-Max	-		-	-	[2.0;4.0]	[4.0;4.0]	[2.0;6.0]	[2.0;6.0]		
														
DOC+EPI+CYC	Missing	-		0	-	-	-		-			0	W = NA	
												
	Mean (Sd)	-		6.00 (0.00)	-	-	-		-	6.00 (0.00)		
												
	Min-Max	-		[6.0;6.0]	-	-	-		-	[6.0;6.0]		
														
DOX+CYC	Missing	-		-	0	-	-		-			0	W = NA	
												
	Mean (Sd)	-		-	3.75 (0.50)	-	-		-	3.75 (0.50)		
												
	Min-Max	-		-	[3.0;4.0]	-	-		-	[3.0;4.0]		
														
EPI	Missing	0		-	-	-	-		-			0	W = NA	
													
	Mean (Sd)	1.00	(--)	-	-	-	-		-		1.00 (--)		
											
	Min-Max	[1.0;1.0]	-	-	-	-		-	[1.0;1.0]		
														
EPI+CYC	Missing	-		-	0	-	0		-			0	T = 9.8	
														

	Mean (Sd)	-	-	4.50 (1.00)	-	1.00	(--)	-		3.80 (1.79)		
											
	Min-Max	-	-	[4.0;6.0]	-	[1.0;1.0]	-		[1.0;6.0]		
												
EPI+FLU	Missing	-	-	-	-	-		0		0	W = NA	
												
	Mean (Sd)	-	-	-	-	-		1.00	(--)	1.00 (--)		
											
	Min-Max	-	-	-	-	-		[1.0;1.0]	[1.0;1.0]		
												
EPI+CYC+FLU	Missing	0	0	0	0	0		0		0	T =	
											76.011	
										
	Mean (Sd)	3.04 (0.45)	6.00 (0.00)	3.50 (1.07)	3.00 (0.00)	3.38 (0.74)	2.83 (0.41)	3.65 (1.25)		
										
	Min-Max	[2.0;5.0]	[6.0;6.0]	[3.0;6.0]	[3.0;3.0]	[3.0;5.0]	[2.0;3.0]	[2.0;6.0]		
												
PAC	Missing	-	-	-	0	-		0		0	W = 0.00	
												
	Mean (Sd)	-	-	-	2.00 (0.00)	-		2.00	(--)	2.00 (0.00)		
											
	Min-Max	-	-	-	[2.0;2.0]	-		[2.0;2.0]	[2.0;2.0]		
												
PAC+CYC	Missing	-	-	-	0	-		-		0	W = NA	
												
	Mean (Sd)	-	-	-	2.00 (--)	-		-		2.00 (--)		
												
	Min-Max	-	-	-	[2.0;2.0]	-		-		[2.0;2.0]		
												
Total number of cycle	Missing	0	0	0	0	0		0		0	W = 35.33	
										
	Mean (Sd)	6.00 (0.00)	6.00 (0.00)	4.75 (1.18)	5.29 (0.96)	6.00 (1.00)	4.67 (1.19)	5.44 (1.01)		
										
	Min-Max	[6.0;6.0]	[6.0;6.0]	[3.0;6.0]	[4.0;6.0]	[4.0;8.0]	[2.0;6.0]	[2.0;8.0]		
												
DOC=Docetaxel; DOX=Doxorubicine; CY= Cyclophosphamide; EP=Epirubicine; PA=Paclitaxel


Table B19. Distribution of cycle and reason for symptomatic prescription

					Drôme							
		Pôle Lille	Paul D'Egine	Clairval	Ardèche	Peupliers	Jean Mermoz		Total	
													
Antieme	Missing	0	0	0		0	0		0			0	
tic													
										
	Yes	150 (100.00%)	102 (100.00%)	76 (100.00%)	64 (57.66%)	54 (100.00%)	84 (100.00%)	530	(91.85%)	
											
	No	0	0	0	47 (42.34%)	0		0	47 (	8.15%)	
													
Growth	Missing	0	0	0		0	0		0			0	
factor													
											
	Yes	95 (63.33%)	64 (62.75%)	64 (84.21%)	2 (	1.80%)	32 (59.26%)	80 (95.24%)	337	(58.41%)	
											
	No	55 (36.67%)	38 (37.25%)	12 (15.79%)	109 (98.20%)	22 (40.74%)	4 (	4.76%)	240	(41.59%)	
													
Anti-hist	Missing	0	0	0		0	0		0			0	
aminic													
												
	Yes	24 (16.00%)	0	0		0	0		0	24 (	4.16%)	
										
	No	126 (84.00%)	102 (100.00%)	76 (100.00%)	111 (100.00%)	54 (100.00%)	84 (100.00%)	553	(95.84%)	
													
Gastro	Missing	0	0	0		0	0		0			0	
mucositi													
s													
												
	Yes	29 (19.33%)	0	0	5 (	4.50%)	0	2 (	2.38%)	36 (	6.24%)	
										
	No	121 (80.67%)	102 (100.00%)	76 (100.00%)	106 (95.50%)	54 (100.00%)	82 (97.62%)	541	(93.76%)	
													
Cardioto	Missing	0	0	0		0	0		0			0	
xicity													
											
	Yes	2 (1.33%)	67 (65.69%)	3 (3.95%)		0	7 (12.96%)		0	79 (13.69%)	
										
	No	148 (98.67%)	35 (34.31%)	73 (96.05%)	111 (100.00%)	47 (87.04%)	84 (100.00%)	498	(86.31%)	
													
Paresth	Missing	0	0	0		0	0		0			0	
esia													
hand													
and foot													
													
	Yes	28 (18.67%)	0	0	0	0	0	28 (	4.85%)	
										
	No	122 (81.33%)	102 (100.00%)	76 (100.00%)	111 (100.00%)	54 (100.00%)	84 (100.00%)	549	(95.15%)	
											
Other	Missing	0	0	0	0	0	0			0	
										
	Yes	9 (6.00%)	0	21 (27.63%)	0	1 (1.85%)	4 (4.76%)	35 (	6.07%)	
										
	No	141 (94.00%)	102 (100.00%)	55 (72.37%)	111 (100.00%)	53 (98.15%)	80 (95.24%)	542	(93.93%)	
											


Table B20. Transportation and distribution of transport

		Pôle Lille	Paul D'Egine	Clairval	Drôme Ardèche	Peupliers	Jean Mermoz		total	
											
Type of transport	Missing	0	0		0	0	0	0		0	
											
	Valid values	297	113		80	154	55	107		806	
											
	Ambulance	0	18 (15.93%)		0	0	0	0	18 (	2.23%)	
											
	VSL	0	36 (31.86%)		0	0	0	0	36 (	4.47%)	
									
	Taxi	0	18 (15.93%)	73 (91.25%)	39 (25.32%)	0	0	130 (16.13%)	
										
	Personal	297 (100.00%)	41 (36.28%)	7 (	8.75%)	115 (74.68%)	55 (100.00%)	107 (100.00%)	622 (77.17%)	
	vehicle										
											
Transport	Missing	0	0		0	0	0	0		0	
voucher											
											
	Valid values	297	113		80	154	55	107		806	
									
	Yes	0	66 (58.41%)	79 (98.75%)	39 (25.32%)	0	0	184 (22.83%)	
										
	No	297 (100.00%)	47 (41.59%)	1 (	1.25%)	115 (74.68%)	55 (100.00%)	107 (100.00%)	622 (77.17%)	
											
